# Skipping of Exons by Premature Termination of Transcription and Alternative Splicing within Intron-5 of the Sheep SCF Gene: A Novel Splice Variant

**DOI:** 10.1371/journal.pone.0038657

**Published:** 2012-06-15

**Authors:** Siva Arumugam Saravanaperumal, Dario Pediconi, Carlo Renieri, Antonietta La Terza

**Affiliations:** School of Environmental Sciences, University of Camerino, via Gentile III da Varano, Camerino (MC), Italy; Argonne National Laboratory, United States of America

## Abstract

Stem cell factor (SCF) is a growth factor, essential for haemopoiesis, mast cell development and melanogenesis. In the hematopoietic microenvironment (HM), SCF is produced either as a membrane-bound (−) or soluble (+) forms. Skin expression of SCF stimulates melanocyte migration, proliferation, differentiation, and survival. We report for the first time, a novel mRNA splice variant of SCF from the skin of white merino sheep via cloning and sequencing. Reverse transcriptase (RT)-PCR and molecular prediction revealed two different cDNA products of SCF. Full-length cDNA libraries were enriched by the method of rapid amplification of cDNA ends (RACE-PCR). Nucleotide sequencing and molecular prediction revealed that the primary 1519 base pair (bp) cDNA encodes a precursor protein of 274 amino acids (aa), commonly known as ‘soluble’ isoform. In contrast, the shorter (835 and/or 725 bp) cDNA was found to be a ‘novel’ mRNA splice variant. It contains an open reading frame (ORF) corresponding to a truncated protein of 181 aa (*vs* 245 aa) with an unique C-terminus lacking the primary proteolytic segment (28 aa) right after the D^175^G site which is necessary to produce ‘soluble’ form of SCF. This alternative splice (AS) variant was explained by the complete nucleotide sequencing of splice junction covering exon 5-intron (5)-exon 6 (948 bp) with a premature termination codon (PTC) whereby exons 6 to 9/10 are skipped (Cassette Exon, CE 6–9/10). We also demonstrated that the Northern blot analysis at transcript level is mediated via an intron-5 splicing event. Our data refine the structure of SCF gene; clarify the presence (+) and/or absence (−) of primary proteolytic-cleavage site specific SCF splice variants. This work provides a basis for understanding the functional role and regulation of SCF in hair follicle melanogenesis in sheep beyond what was known in mice, humans and other mammals.

## Introduction

Many growth factors such as colony-stimulating factor-1 (CSF), transforming growth factor- α (TGF-α), and tumor necrosis factor (TNF) occur in both membrane-bound and secreted forms [1] by specific proteolytic cleavages. These growth factors and their receptors play vital roles in normal development as mediators of intercellular communication by diffusible molecules and often promote cell differentiation and maturation. Stem cell factor (SCF) [2] also known as steel factor (SLF or SF) [1], [3]; mast cell growth factor (MGF) [4], [5]; kit ligand (Kitl, KL or KITLG) [6] is one of several pleiotropic growth factors, a cytokine that binds to its cognate c-KIT receptor or stem cell factor receptor (SCFR) [2], the product of the *c-kit* gene. SCF is encoded by the murine *Steel* (*Sl*) locus while KIT is encoded by *dominant white spotting* (*W*) *KIT* locus in the mouse [7], [8]. SCF plays an important role in hematopoiesis, spermatogenesis, and melanogenesis [1]. In the hematopoietic microenvironment (HM), SCF is produced either as a membrane-bound or soluble form [9], [10].

SCF is produced as transmembrane proteins that are released by specific proteolytic cleavage to generate soluble factors [3], [5], [11]. Alteration in the balance between the diffusible and membrane-bound forms may lead to phenotypic abnormalities as previously reported in *dominant white spotting* (*W*) or the *Steel* (*Sl*) loci which are among the most studied mutations in mouse [7], [8], [12]–[15]. Investigations into the expression of c-KIT and SCF in the skin during melanocyte migration are consistent with the known *W* and *Sl* phenotypes and suggest that SCF mediates a chemotactic/hapatotactic signal for c-*kit* in the development of pigmentation [6]. The membrane-bound SCF/c-KIT signalling could act on mammalian hair follicle melanogenesis during cyclic anagen phases, resulting in hair follicle pigmentation [16]. Besides its role as a melanocyte survival factor, SCF can also act synergistically with several interleukins and granulocyte-macrophage-colony stimulating factor to enhance UV-induced pigmentation [17], [18]. The signalling of SCF and its receptor c-KIT has been documented to regulate essential roles in the maintenance of embryonic melanocyte lineages and postnatal cutaneous melanogenesis [3], [19]–[21].

Alternative Splicing (AS) is a key element in gene regulation that increases proteome diversity and the coding potential of various eukaryotic genomes. Evidence from expressed sequence tags (ESTs), cDNA, genome-wide Tilling and splicing microarray datasets in human demonstrate that alternative splicing occurs in 90% of genes [22], [23]. The high incidence of AS in the pigmentation gene network for example SCF/c-KIT [14], [16] and MITF [24] might contribute to the regulation of their switch in the development of various genetic disorders and phenotypic abnormalities. In the case of SCF gene, AS results in two membrane-bound protein products [5], [6], [25]. To date, two alternatively spliced forms of SCF mRNA have been reported in the mouse: 1) the full-length form; and 2) an alternative form lacking exon 6, which like the corresponding human transcript, produces a 28 aa deletion. Exon 6 codes for an extracellular cleavage site, which is susceptible to proteolytic cleavage by proteases. Expression of the SCF variant containing this exon 6 will produce a membrane-bound isoform, designated as SCF-1 (KL-1) or (+) form, and its proteolytic cleavage will generate a soluble form of the factor. In contrast, expression of the SCF splice variant, lacking exon 6, gives rise to a stringent membrane-bound protein, known as SCF-2 (KL-2) or (−) form [5], [6], [11], [25]. The SCF expression ratio between the KL-1 and KL-2 isoforms varies significantly between various cell types [6], [11]. A Mouse mutagenesis study [10] reported the usage of secondary cleavage site in the absence of primary cleavage site (exon 6) to generate the soluble form and is located at or near Lys^178^-Ala^179^-Ala^180^-Ser^181^ (exon 7).

Three isoforms have been identified and documented for human and mouse SCF genes (source: GenBank, NCBI, http://www.ncbi.nlm.nih.gov/; Ensembl, www.ensembl.org/; UniProt, www.uniprot.org/). Basically, the first two isoforms (273 aa and 245 aa) differ by the presence (+) and absence (−) of potential primary proteolytic site (exon 6), respectively. The third, shortest isoform (238 aa) differ in its N-terminus for the first 8 aa *vs* first 43 aa of the (+) and (−) form but has the primary proteolytic site. In sheep, there exists only two partial mRNA records, the counterparts of SCF-1 (+ form), one from ovarian follicle (780 bp, Acc. No. U89874.1), the other in keratinocyte (622 bp, Acc. No. Z50743.1) and two partial records of SCF genomic DNA sequences i.e., a 5′ UTR and partial CDS sequence (358 bp, Acc. No. HM347344.1), the stem cell factor MGF25 (781 bp, Acc. No. AF165788.1) gene, coding region not determined. The larger mRNA species of SCF encodes a protein (Uniprot, P79368) of 267 amino acids (aa), known as ‘soluble’ isoform (SCF-1/b), which is a transmembrane protein comprising of a 25 aa leader signal peptide sequence, a 189 aa extracellular domain that includes a proteolytic cleavage site (28 aa), followed by a hydrophobic membrane spanning helical region (21–23 aa) and a short cytoplasmic tail (36–37 aa) [5], [6], [25]. The alternative SCF mRNA lacks exon 6, a deletion of 84 bp. This shorter mRNA species gives rise to a protein, known as ‘membrane-bound’ isoform (SCF-2/a) that lacks 28 aa, including one of the four N-linked glycosylation sites in the C-terminus (Ala^164^ and Ala^165^) of the soluble SCF, as well as the protease recognition site. This shorter form of the protein yields soluble SCF less efficiently than the longer form of the transmembrane protein. Hence the regulation of the abundance of the alternatively spliced messages might significantly contribute to the regulation of the production of soluble and/or membrane-associated SCF by the cell [26]. The physiologic roles of these SCF proteins remain uncertain. Notably, the biological effects of the membrane-bound (as opposed to soluble) forms of the protein may be significantly different, at least with respect to bone marrow progenitor cells [9].

Numerous pigmentation mutants are phenotypically (>800 alleles) profound, but remain mechanistically uncharacterized [27]. In sheep, the candidate genes for recessive black (ASIP) [28], [29], dominat black (MC1R) [30], [31] and Brown (*tyrosinase related protein-1*, TYRP1) [32] have been found which are known to influence pigmentation or pigment synthesis level. In the merino experimental models [33], authors proposed that “*The inheritance of white coat colour in merino sheep is dependent on single gene segregation, without any modifying effects and is completely dominant over pigmented animals*”. According to their data, *Agouti* (*A*) locus or *extension* (*E^D^*) locus [28]–[31] which are encoded by *agouti signalling peptide* (ASIP) and the *melanocortin-1 receptor* (MC1R) loci respectively [34] have never been associated with spotting or white in mammals. They are involved, in fact, in melanin switching [13], [35], [36]. White can be caused by defects at various stages of melanocytes development, including proliferation, survival, migration, invasion of the integument, hair follicle entry and melanocytes stem cell renewal [36]. Many white spotting traits have been identified in mouse and man, and 10 of the genes have been cloned [36]. It has been hypothesized that the gene for white phenotype in merino sheep is on these loci [33]. Among those, for the loci *microphthalmia-associated transcription fact*or (MITF, *microphthalmia*) [37], c-KIT (*Dominant White Spotting*) and SCF (*Steel*), it is possible to obtain completely white live animals [27], [36], [38]. Since c-KIT/SCF signaling and MITF-dependent transcription are both essential for the melanocyte development and pigmentation [39].

The study of genes controlling coat colour and pigmented fibres are most relevant to ‘white’ wool production as brown or black wool will not dye as readily. Since natural coloured fibre is a new opportunity for textile industries, development of valid genetic tools (coat colour tests) and effective sheep breeding programme should go hand-in-hand to help breeders and small scale farmers to reduce future occurrences with the wool market. The present empirical study was undertaken as part of the huge in-house project evaluating the involvement of three candidate genes such as MITF, c-KIT and SCF in various coat colour traits of merino sheep especially the white phenotype. Isolation of these genes and knowledge of their structure will allow for further studies into the regulation of gene expression in the ovine melanocyte biology and skin pigmentation. In an effort, to better characterize the mRNA/cDNA structure of SCF in the skin of white merino sheep we performed cDNA cloning, sequencing and gene expression analysis by semi-quantitative RT-PCR and Northern blot. In this study, we isolated a novel mRNA splice variant from skin designated as ‘SCF *truncated isoform-2a/b* (−)’, demonstrating for the first time, that a premature stop codon (PTC) at the short 3′ UTR sequence corresponding to intron 5 is due to the usage of an alternative splice donor/acceptor site. The other ovine transcript variant, ‘SCF *isofrom-1* (+)’ expressed in skin, the commonly known homolog of SCF (+) isoform in other mammals is also been presented here. We also demonstrated that the relative gene expression at mRNA transcript level is mediated via an intron 5 splicing event by Northern blot analysis. Further, this manuscript discusses extensively on ovine SCF mRNA structural coverage, putative AS events on the intron-5 of the SCF gene, mRNA and protein structure domain characterization, homology modelling, and molecular phylogeny of SCF.

## Materials and Methods

### Collection of Skin Biopsies and Blood

Skin biopsies were collected from uncoloured (white) and coloured (black and brown) animals of the merino sheep using disposable, sterile, biopsy punch (8 mm diameter), treated and stored in RNAlater (Sigma-Aldrich, Milan, Italy), transferred to the molecular biology laboratory and immediately frozen in liquid nitrogen until RNA extraction. Blood samples were collected from the jugular vein of the same individuals with PAXgene Blood DNA Tubes (PreAnalytix kit, Qiagen, Milan, Italy) via standard phlebotomy technique, processed immediately in the lab according to the manufacturer’s protocol for the DNA isolation and the aliquotes were stored at -80^o^C. Samples were collected and recorded according to the farm technicians from Aziende la Campana Montefiore dell’Aso (Ascoli Piceno, Marche), La Meridiana Umbertide (Perugia, Umbria), Italy with permission from the owners of each farm.

### RNA and DNA Isolation and Quantification

Total RNAs were extracted from the stored skin biopsies of all three animals using TRI Reagent (Sigma-Aldrich, Milan, Italy) according to the manufacturer’s instructions followed by treatment with RNase-free DNase (Fermentas, Milan, Italy) to remove contaminated DNAs. Tissue was homogenized (0.075 g in 750 µl TRI reagent) using Polytron homogenizer (Qiagen, Milan, Italy). Genomic DNAs were isolated from the blood samples with PAXgene Blood DNA kit (PreAnalytix kit, Qiagen, Milan, Italy) following the given handbook protocol.

The qualitative assessment of the isolated, purified DNAs, RNAs were done utilizing the Genesys 10 UV Spectrophotometer (Thermo Electron Corporation, Madison, USA). The purity was assessed by calculating the ratio of optical density (OD) at A260/A280 and the integrity was determined by running the samples on 1.0% formaldehyde-agarose gel electrophoresis for RNA and 0.8% agarose gel electrophoresis for DNA [40]. For DNA, the concentration was also evaluated based on the intensities of band with reference to the molecular weight standard Lambda (λ) DNA EcoRI HindIII digest (Fermentas, Milan, Italy) or 1 kb gene ruler (USB Corporation, Cleveland, USA). The nucleic acid concentration was calculated following [40] and the DNA samples were diluted to 10 ng/µl or 50 ng/µl for PCR amplification.

### cDNA Synthesis and RT-PCR Amplification

cDNAs were synthesized from total RNA extracted from the skin of the merino sheep. Reverse Transcription (RT) from 1–1.5 µg of RNA in a toal volume of 20 µl containing 50 pmol oligo(dT) (18-mer) or oligo(dT)_18_ modified primer, 0.5 mM deoxyribonucleoside triphosphate (dNTPs), 1×RT buffer, 20 U of RNase inhibitor and 200 U PrimScript™ Reverse Transcriptase (Takara Bio Inc., Clontech, Jesi, Italy) or StrataScript™ Reverse Transcriptase (Stratagene, Agilent Technologies, Milan, Italy) according to the manufacturer’s instructions. The reaction was incubated for 60 min at 42°C and then heated at 70°C for 15 min, and cooled on ice. All the RT reactions were performed in a Perkin-Elmer Cetus Model 480 DNA Thermal Cycler (Perkin-Elmer, Monza, Italy) and/or MyCycler™ Thermal Cycler (Bio-Rad Laboratories, Segrate, Italy). Subsequently, 0.5–0.7 µl of the first strand cDNA reaction was used for PCR amplification. The reactions were performed in 25 µl volume containing 1×PCR bufffer, 1.5 mM MgCl_2_, 2.0 mM dNTPs, 0.3–0.5 µM gene specific primers (Table S2), 20–30 ng/ul cDNA and 1.5 U of proofreading Easy-A High-Fidelity PCR Cloning Enzyme (Stratagene, Agilent Technologies, Milan, Italy) and the cDNA check amplification was performed with Dream Taq DNA polymerase (Fermentas, Milan, Italy). Three-step RT-PCR amplification was performed in a MyCycler™ Thermal Cycler (Bio-Rad Laboratories, Segrate, Italy), TGRADIENT Thermocycler (Biometra GmbH, Göttingen, Germany) with an initial denaturation at 95°C for 3 min, followed by 5 primary cycles of 94°C for 1 min, annealing temperature (Ta^o^C) below 3–5°C of the temperature melting (Tm) of the gene specific primer whichever is lowest of the two primers for 1 min, and a 72°C for 1 min. This was then followed by 25 consecutive cycles of 94°C for 15–30 sec, annealing temperature (Ta^o^C) for 15–30 sec and 72°C for 20–30 sec with a final extension at 72°C for 10 min, lastly a hold temperature at 4°C. NOTE: PCR cycling conditions especially Ta, timing interval varies with primer sets and the expected size of amplicons (see Table S2 for details).

The isoform (+) specific primer pair for the the amplification of the open reading frame (ORF) corresponding to the 621 bp of the sheep SCF cDNA was designed based on the coding sequence homology among human (GenBank Acc. No. NM_000899.3), chimpanzee (XM_509255.2), marmoset (XM_002752832.1), orangutan (XM_002823566.1), mouse (NM_013598.2), rat (NM_021843.3), panda (XM_002921694.1), cat (NM_001009343.1), horse (NM_001163962.1), dog (NM_001012735.1), goat (AB002152.1), pig (NM_214269.2), cow (NM_174375.2) and sheep (GU386372) using Primer3 software [41]. The remaining 5′ and 3′ RACE SCF gene specific primer pairs were deduced from the 621 bp cDNA coding sequence (CDS) fragment to walk up and down in order obtain the full-length cDNAs. All the designed primer pairs were checked with the online software tools [42], [43] before making an order with [42]. The primers used in this study were synthesized and purchased from Sigma-Aldrich, Milan, Italy.

### Rapid Amplification of cDNA end Experiments (5′ and 3′ RACEs)

We performed 5′ and 3′ RACE experiments to isolate and determine the sheep full-length SCF cDNA(s). This was done following the instructions of 5′ (v. 2.0) and 3′ (v. E) RACE System for Rapid Amplification of cDNA Ends (Invitrogen, Life Technologies, Monza, Italy).

5′ RACE cDNAs were reverse transcribed from 1–1.5 µg of RNA in a total volume of 20 µl containing 2–2.5 pmol SCF gene specific splice variant primers (Table S2), 0.5 mM dNTPs, 1×RT buffer, 20 U of RNase inhibitor, 200 U of PrimScript™ Reverse Transcriptase (Takara Bio Inc., Clontech, Jesi, Italy) and StrataScript™ Reverse Transcriptase (Stratagene, Agilent Technologies, Milan, Italy) according to the manufacturer’s instructions. The reaction was incubated for 60 min at 50°C and then heated at 70°C for 15 min, cooled on ice and stored at −20°C. Two different 5′ RACE cDNAs were synthesied with gene specific primer for the SCF (+) and (−) form (see Table S2 for details). This was then followed by 0.1 volume of 3 M sodium acetate, pH 4.8 or 5.2 salt and 2.5 volume of 100% ethanol preicipitation to the final volume of 100 µl 5′ RACE cDNA. The precipitation was carried out at −80°C over night and centifuged twice at 16,000 g for 30 min. The pellet was washed twice with 70% ethanol at 16,000 g for 15 min. The collected, air dried pellet was finally dissolved in 40 µl DEPC treated water and stored as aliquotes at −80°C. A homopolymeric tail was then added to the 3′-end of the purified cDNA (10 µl) using 30 U terminal deoxynucleotidyl transferase (TdT, USB Corporation, Cleveland, USA and Invitrogen, Life Technologies, Monza, Italy) and 0.2 mM dCTP (Fermentas, Milan, Italy) following the protocol of 5′ RACE System (v. 2.0, Invitrogen, Life Technologies, Monza, Italy). The reaction was incubated at 95°C for 3 min for the denaturation and 37°C for 12 min for the addition and then heat inactivated at 70°C for 10 min, cooled on ice and stored at −20°C. Subsequently, 3 µl of the dC-tailed cDNA was used in a final volume of 50 µl for the first round enrichment PCR amplification followed by second round nested amplification using 2–3 µl of the primary enriched RT-PCR reaction. The primer combinations used were adapter forward primers aapfwd (first round), auapfwdnst (second round, nested) and scfrev1 (proteolytic site, + form, first round), scfrev2 (common region, - form, first round) and scfrev3 (common, second round) as the reverse primers, respectively. NOTE: Forward adapter primer sequences were retrieved from the 5′ RACE kit, Invitrogen, Life Technologies, Monza, Italy and synthesised by Sigma-Aldrich, Milan, Italy. The PCR amplification was carried out as described above for 36 cycles and the cycling conditions especially Ta, timing interval varies with 5′ RACE primer sets and the expected size of amplicons (see Table S2 for details).

First strand 3′ RACE cDNAs were prepared with a high Tm oligo(dT)_18_ modified primer as described above and 1 µl of this cDNA was used in a final volume of 50 µl for the first round PCR amplification. Successive nested, splice variant specific amplifications were performed in a 50 µl PCR volume using 1 µl of the primary enriched RT-PCR reaction.

The PCR was run for 36 cycles as described above and the cycling conditions especially Ta, timing interval varies with specific 5′ and 3′ RACE primer sets (see Table S2 for details). For 3′ RACE the primer pairs having high Tm were subjected to a two-step PCR with a coupled annealing, extension at 69 or 72°C for 3 min 10 sec up to 10 min. The primer combinations used for distinctive 5′ and 3′ RACE amplification and the expected size of amplicons were presented in Table S2. NOTE: Forward adapter primer sequences were retrieved from the 5′ RACE kit, Invitrogen, Life Technologies, Monza, Italy and synthesised by Sigma-Aldrich, Milan, Italy.

### DNA Splice Junction Amplification

Blood genomic DNA was amplified to confirm the splice site premature termination with a poly A signal detected on sheep SCF cDNA transcripts. The Expand Long Range, dNTPack (Roche S.p.A., Milan, Italy) was used following the manufacturer’s instructions, including 0.3–0.5 µM specific primers scffwd3 (exon 5, common) and scfrev1 (exon 6, + form specific) (Table S2), 500 µM dNTP mix, 3% DMSO, 100–150 ng of genomic DNA and 3.5 U of Expand Long Range Enzyme mix in a final 50 µl PCR volume. The PCR protocol was performed as per Roche’s kit protocol. Since the available unfinished draft reference sheep genome, Oarv2.0 (current version, March 2011 - till date, http://www.livestockgenomics.csiro.au/sheep/oar2.0.php) did not provide much information regarding the SCF gene, the reference SCF genomic locus at the exon 5-intron (5)-exon 6 splice junction was covered in comparison to the orthologous SCF gene assembly of human, mouse, cow and dog.

### Expression of Ovine SCF in Skin

To determine the relative abundance of the SCF (+) and (−) cDNA transcripts, we performed semi-quantitative RT-PCR amplification using two different sets of splice variant specific (+ and -) primers as summarised in Table S2. Four sets of primer pair included (+) form specific forward (exon 5-exon 6: stpro3’Rfwd1), reverse primer (exon 6: scfrev1); the common forward primers (scffwd1, scffwd4) located on the common region of the CDS and a (−) form specific reverse primer (scf(−)rev) which was designed spanning into the exon 7-exon 5 splice junction. Total RNA of 1.5 µg of each animal (white, black, brown) was reverse transcribed into cDNA using 200 U PrimScriptTM Reverse Transcriptase (Takara Bio Inc., Clontech, Jesi, Italy) and 50 pmol oligo(dT) modified primer in a 20 µl reaction volume, as described above. PCR amplification was performed using 0.5 µl of the each cDNA sample as a template in 25 µl of a reaction mixture consisting of 1×DreamTaq buffer, 1.5 mM MgCl_2_, 0.2 mM dNTPs, 0.5 µM each of primer and 1.5 U of DreamTaq DNA polymerase (Fermentas, Milan, Italy). After an initial denaturation step of 3 min at 95°C, a 3-step PCR programme was carried out with 5 successive cycles of 25 sec at 95°C for denaturation, 25 sec at primer-specific annealing temperature (Ta°C) for annealing procedure and 25 sec at 72°C for extension, followed by 25 repeat cycles of 94°C for 15 sec, annealing temperature (Ta^o^C, see Table S2) for 15 sec and 72°C for 20 sec with a final extension at 72°C for 10 min and a cooling phase at 4°C. Amplified RT-PCR products were separated on 1.5–2% agarose gel electrophoresis, and were evaluated by ethidium bromide staining and UV transillumination. For the RT-PCR reference, constitutively expressed glyceraldehyde 3-phosphate dehydrogenase (GAPDH, 252 bp) and 18 S rRNA (132 bp) was used as an equal loading control. The house keeping gene (HKGs) primers were designed from the corresponding *Ovis aries* NCBI GenBank Accession Nos. (see Table S2) and amplified with the same PCR conditions and cycle numbers. Amplicons were confirmed by cloning and direct sequencing. The relative signal strength was measured using the QuantiScan Demo software [44].

For Northern blot analysis, total RNA was isolated from skin as described above. The poly(A)+ mRNA from total RNA was purified using Oligotex mRNA Midi Kit (Qiagen, Milan, Italy) following the manufacturer’s protocol. The 40 µl eluted mRNA sample was separated on a 1.2% denaturing formaldehyde-agarose gel electrophoresis [40]. Subsequently, mRNA was transferred to a Hybond™-N Neutral nylon membrane (Amersham Biosciences, GE Healthcare Europe GmbH, Milan, Italy) overnight by capillary diffusion [40]. The mRNA was crosslinked onto the membrane by baking at 80°C for 2 h. The membrane was pre-hybridized at 50°C for 1 h and then hybridized overnight at 50°C containing denatured DIG-labeled PCR probe (2 µl/ml). DIG-labeled PCR probes were synthesized using a PCR DIG Probe Synthesis kit (Roche S.p.A., Milan, Italy). DIG labeled DNA fragments of ovine SCF (222 bp, +/− form) and 18 S rRNA (132 bp) were synthesized by PCR using the corresponding cDNA clones as templates and gene-specific primers (see Table S2). Following low (2×5 min with 2×SSC, 0.1% SDS at room temperature) and high (2×15 min with 0.1×SSC, 0.1% SDS at 50°C) stringent washes, the nylon membrane was incubated in the blocking solution for 45 min followed by additional incubation with a blocking solution that contained a 1∶5,000 dilution of alkaline phosphatase conjugated, anti-DIG antibody (Roche S.p.A., Milan, Italy) and incubated for 15–45 min at room temperature. The hybridized probe was detected with the chemiluminescent substrate, CSPD (Roche S.p.A., Milan, Italy). Hybridization signals were detected by exposure of the membrane to Kodak® BioMax® XAR Film (Sigma, Milan, Italy) at room temperature. Pre-hybridization, hybridization, blocking and washing solution recipes were prepared and followed according to the procedures for nonradioactive (DIG) labeling and detection of nucleic acids (Roche S.p.A., Milan, Italy). Probes were stripped at 80°C for 2×60 min before rehybridization according to the manufacture’s instructions (DIG application manual, Roche S.p.A., Milan, Italy).

### Gel Electrophoresis and Photography

Amplified products were subjected to 1.2–1.5% agarose gel electrophoresis using 1×TAE buffer (40 mM Tris-acetate, pH 8.0, 1 mM EDTA) at 5–7 V/cm. The gels were stained with 0.5 mg/ml ethidium bromide, visualized on ultraviolet transilluminator (Macrovue model 2011, LKB Produkter, Bromma, Sweden). Gels were captured and analyzed using Kodak Digital Science DC40, 1D software for Electrophoresis Documentation and Analysis System (Kodak, Rochester, New York, USA).

### Cloning and Sequencing

All the selected amplicons were gel purified either manually by salt precipitation or using Nucleospin columns (Macherey-Nagel, GmbH & Co. KG, Düren, Germany). Cloning was performed in the TA cloning system (pGEM®-T Easy, Promega, Milan, Italy; pCR®2.1 TOPO, Invitrogen, Life Technologies, Monza, Italy; InsTAclone™, Fermentas, Milan, Italy and pSC-A, StrataClone-UA, Stratagene, Agilent Technologies, Milan, Italy). The ligated products (3–5 µl) were transfered by heat shock treatment into a chemically competent DH5α cells which were prepared manually [40], except for pSC-AStrataClone-UA vector system for which StrataClone SoloPack competent cells were used (included in the kit package). Clones were screened by M13 colony PCR amplification. Identified positive colonies were inoculated into the selective antibiotic LB or SOB medium for the over night culture at 37°C, 150 rpm in a shaker waterbath. Subsequently, plasmid DNAs were isolated [40] and screened for the release of expected insert(s) by analytical single or double restriction enzyme digestion (EcoRI or EcoRI+HindIII) according to the vector map. Positive clones were prepared for sequencing and sequenced by the commercial vendors (StarSEQ, Mainz, Germany; BMR sequencing, Padova, Italy) with M13 forward and/or reverse primer or sequenced with any one of the gene specific primer for deeper sequencing of the inserts whenever necessary. Sequences were viewed with sequencing chromatogram trace viewer FinchTV v. 1.4.0 [45].

### Sequence Data

Our new sequenced data of SCF can be accesed through NCBI GenBank accession nos. GU386371– GU386374 (Table S1).

### mRNA Secondary Structure Analysis

We used the webserver program Mfold v. 3.5 [46] for predicting the non-coding RNA (ncRNA) secondary structure stability of the different SCF transcripts and its miRNA target binding sites. The structure of DNA splice junction was analysed with DNA Folding Form [46]. The ncRNA secondary structures were also predicted with a set of MUSCLE [47] aligned mammalian homologous sequences of the SCF cDNA transcripts using Sequences Selection for the Comparative Approach (SSCA) by Tfold [48]. The optimal secondary structures for all sequences were obtained in a dot-bracket notation with minimum free energy and the structural elements such as helices, internal and terminal loops were deterrmined by drawing the RNA structure in the java applet VARNA v. 3.7 [49]. All fold analyses were performed using the default setting of the web servers.

The TargetScan program Release 5.1 [50] and miRBase Release 16 [51] were used to locate potential sheep SCF 3′ UTR miRNA target sites from human, mouse, dog, cow and chicken.

### Protein Homology Modelling

Protein templates were identified and scrutinized using Template Identification tool at SWISSMODEL Workspace v 8.0.5 [52], Reverse PSI-BLAST (in BLAST 2.2.12 packages) search against protein data bank (PDB) and Structural Classification of Proteins (SCOP) at Genomes TO Protein structures and functions (GTOP) [53].

The homology modeling was performed with Modeller 9v2 [54] using an integrated multiple sequence alignment and multiple structure visualization application ‘Friend’ v. 2.0 [55]. All the modelled structures were stored as a PDB format data (.pdb) and then viewed, edited with ViewerLite v. 5.0, Discovery Studio Visualizer 2.5.5 [56]. Modelled structures were assessed with Protein Structure and Model Assessment Tools at SWISSMODEL Workspace. The secondary structures such as Alpha helix, Beta strand, Beta bulge, 3,4,5-turns were defined with respective colours using CCP4MG release 2.4.3 [57]. Homology modeling was also attempted with an automated modeling server at SWISSMODEL Workspace [52].

### Sequence Analysis and Molecular Phylogeny

Whole mammalian genome scanning was done to identify the homologous regions of sheep SCF cDNA transcript variants using Basic Local Alignment Search Tool (BLAST) at National Center for Biotechnology Information (NCBI), Bethesda, Maryland, USA [58], ENSEMBL release 60 [59] and BLAT [60] searches, sequentially. Sequences were edited, translated using the BioEdit v.7.0.5.2 (Ibis Therapeutics, Carlsbad, CA, USA) [61] and DNASTAR 7 [62] software packages. The open reading frame (ORF) of the full-length SCF cDNAs was determined by DNASTAR 7 [62] and ORF Finder at NCBI (www.ncbi.nlm.nih.gov/gorf/). The positions of exons and introns were determined and the translated SCF protein to genome structure was drawn using WebScipio [63] in reference to the SCF gene structure of human, mouse and dog. ClustalW2 [64] and MUSCLE [47] programs were used to align the DNA and protein sequences. Subsequently, Gblocks program [65] was used to eliminate the poorly aligned positions and divergent regions on the DNA and protein alignments for the phylogenetic analysis. The datas were then converted to FASTA (.fas) and NEXUS (.nex) formats using DataConvert (v. 1.0) [66].

Distance based neighbour-joining (NJ) phylogenetic trees were generated using the Molecular Evolutionary Genetics Analysis (MEGA) software v. 4.1 [67]. The NJ algorithm [68] was implemented with the p-distance [69], Jukes-Cantor [70] and Tamura-Nei [71], [72] model using a transition+transversion substitution at uniform rates as well with the gamma parameter of 4.0. The robustness of each phylogeny was assessed by percentage of 1000 bootstrap (BS) [73] re-samplings.

Phylogenetic relationships were inferred using Maximum Likelihood (ML) method with the programs PhyML-aLRT (v. 2.4.5) [74], RAxML (v. 2.2.3) [75] using the java application program TOPALi (v. 2.5) [76] and MrBayes (v 3.1.2) [77] for the Bayesian Inference (BI) analyses. Among the 88 models tested, two best models Hasegawa Kishino Yano (HKY) [78] plus gamma (+G) distributed rate heterogeneity, General Time-Reversible (GTR+G) [79] matrices for nucleotides and Jones Taylor Thornton (JTT+G) [80] matrix for protein alignments were chosen and subjected to ML analyses as described above. The topology of the trees was inferred by running 1000 bootstrap replicates and expressed as a percentage.

Bayesian Inference consisted of two independent Markov Chain Monte Carlo (MCMC, mcmc nruns) runs of 100,000 (ngen) were calculated with trees samples at every 10^th^ generation and with a prior burn-in of 25% (sump burnin = 2500; sumt burnin = 2500) i.e., the first 2500 sampled trees were discarded. BI was run with GTR+G, HKY+G and a JTT+G substitution models under the above set parameters for the nucleotide and amino acids alignments, respectively.

Molecular phylogeny models were selected based on the *Akaike Information Criterion* (AIC), *Akaike Information Criterion corrected* verion (AICc), *Bayesian Information Criterion* (BIC) and/or *log Likelihood* (-lnL) scores, implemented in jModeltest (v. 0.1.1) [81] for nucleotides and ProtTest (v. 2.4) [82] for proteins. Models selection were also performed and compared with TOPALi (v. 2.5) [76]. All the tree files (NJ, ML, BI) were stored in Nexus (.nex) or New Hampshire Tree (.tre) format. Trees were inspected and prepared in FigTree v 1.3.1 software [83].

### Use of other Computational Tools and Databases

Ovine SCF transcripts were searched on chr. 5 of the *Bos taurus* (Btau_5.2, current release 2011) chromosomal map using NCBI map Viewer [84]. The sequence similarity was visualized with Circos table viewer [85]. The post-transcriptional associated regulatory elements located in the 5′ and 3′ untranslated regions (UTRs) of the SCF cDNA transcripts were retrieved from UTR databases (UTRdb or UTRSite) [86] using the online tools UTRScan and UTRBlast. The graphical representation of SCF amino acid and nucleic acid multiple sequence alignment was drawn by a sequence logo generator, WebLogo (v. 2.8.2) [87]. SCF polyadenylation sites were predicted using the polyADQ web server [88]. Alternative splicing pattern of the ovine SCF transcripts with human, mouse reference assembly were predicted using ACEVIEW [89] and Alternative Splicing and Transcript Diversity (ASTD 1.1) [90]. The splice site prediction such as putative alternative exon isoform, cryptic and constitutive splice sites of internal (coding) exons was performed using Alternative Splice Site Predictor (ASSP) [91] and Regulatory RNA Motifs and Elements Finder (RegRNA Release 1.0) [92]. SCF protein knowledge, sequence analysis, classification were performed with the UniProtKB Protein existence Server [93]. SCF protein secondary structure and site interactions were analyzed using Protein data Bank (PDB) [94] and PDBsum [95]. The putative SCF protein domain figure was drawn with MyDomains - Image Creator at ExPASy [96].

### Ethics Statement

In agreement with the new European Directive on the protection of animals used for scientific purposes (Directive 2010/63/EU, Article 15, Annex VIII), all animal procedures used in the study are classified as ‘mild’ (i.e. procedures with no significant impairment of the well-being or general condition of the animals) and have been preemptively approved by the Animal Ethics Committee of the University of Camerino.

## Results

### Identification and Isolation of the Sheep SCF cDNA Fragment

**Figure 1:**
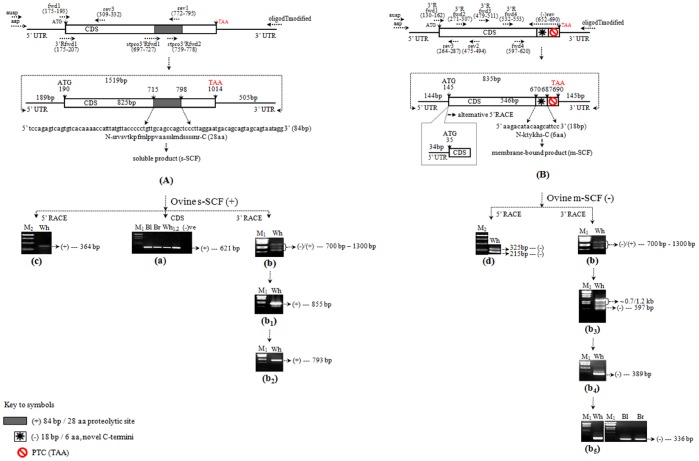
Schematic illustration of primer walking strategy for the ovine SCF (oSCF) mRNA/cDNA transcripts from skin. Gel pictures show subsequent RT-PCR and RACE amplification of the resultant full-length structural coverage of s-SCF (+) and m-SCF (−). The (+) and (−) product is indicated with two different symbols (see key to symbols). In the above figure, the arrows indicate the corresponding position of fwd and rev primers and the split regions of 5′ UTRs, CDS and 3′ UTRs are labeled with respective positions and base pairs (bp). The start and stop codon is labeled in ‘black’ (ATG) and ‘red’ (TAA) letters repectively. (**A**) **Illustration for the full-length cDNA coverage of ovine s-SCF (+)** ***isofom-1***. (**a**) Amplification of isoform specific coding region (CDS) of ovine s-SCF (+) cDNA fragment (621 bp). Individual animal of merino sheep such as Black, Brown, White and the PCR negative control is indicated as Bl, Br, Wh_1,2_ (two individuals) and (−)ve, respectively; (**b**) 3′ RACE first round amplification of oSCF common region (+/−) showing three different sizes of amplicon ranging from ∼700 bp to 1300 bp; (**b_1_**) Isoform specific second round (Nested 1) 3′ RACE of ovine s-SCF (+) cDNA fragment (855 bp); (**b_2_**) Gel picture shows the purified 3′ RACE product of 793 bp (Nested 2 amplification); (**c**) 5′ RACE proteolytic site specific amplification (364 bp) of ovine s-SCF (+). (**B**) **Illustration for the full-length cDNA coverage of ovine m-SCF (**−**)** ***isoform-2***. A premature termination codon (PTC) is indicated in red symbol and the resultant alternative open reading frame (ORF) responsible for the shorter truncated product is highlighted in black open box symbol; (**b_3_**) 3′ RACE amplification (Nested-1) from (b) indicates a 597 bp ovine m-SCF (−) amplicon and the other non-specific products (∼0.7/1.2 kb); (**b_4_**) Further, Nested 2 amplification yielded a 389 bp ovine m-SCF (−) amplicon; **(b_5_**) Gel picture shows the Nested 3 amplification of a 336 bp ovine m-SCF (−) product amplified from either (b_3_) or (b_4_) or (b) or directly from the oligo(dT)_18_ modified primed cDNA; **(d**) 5′ RACE amplification of the common region (+/−) showing two oSCF cDNA products (325 and 215 bp) characterized as ovine m-SCF (−) *isoform2a/ab*, respectively. DNA size markers are indicated as, **M_1_**– λ-DNA EcoRI/HindIII digest; and **M_2_** - 1 kb Gene Ruler. In the above figure, the arrow marks indicate the appropriate size(s) of amplicon of the respective (RT)-PCR amplification. Note: For simplification we removed the tag ‘scf’ from the primer notation (see. Table S2).

To examine the SCF variant(s) expressed in the skin of white merino sheep, 1–1.5 µg of total RNAs from the skin were reverse transcribed and the synthesized single strand cDNAs were amplified by PCR. We initially carried out the cDNA coding (CDS) region amplification using the primer pair scffwd1 and scfrev1 (Table S2). Primer walking and the mRNA/cDNA structural coverage of the longer and shorter cDNA amplification strategies from the ovine total RNA (skin) are shown in Figure 1A and 1B. RT-PCR primers were selected based on the mammalian nucleotide (nt) sequence alignment of the soluble-SCF (s-SCF) cDNA encompassed to the open reading frame (ORF) of 606 bp of the 621 bp amplicon (Figure 1A(a)) commonly known as ‘soluble or secreted form’. The purified RT-PCR amplification product was then cloned and sequenced. Sequencing results revealed no differences among white, black and brown clones of the 621 bp (Figure S1A), which additionally appear to be identical (99%) with two of the previously submitted NCBI GenBank mRNA (partial) sequences of ovine s-SCF (U89874.1 in 2002 and Z50743.1 in 2005; see Figure S1B) from ovarian follicles and keratinocytes, respectively. An exception of transition at T^54^C in U89874.1 was observed among the 621 bp sequences. Similarly, a transversion at C^81^G was observed (see the chromatogram of Figure S1B) in 2 out of 5 clones sequenced in white animal. The possible allelic variant at this position will elucidate its true identity. Nevertheless, these substitutions do not result in an amino acid substitution change.

The virtual translation of the 606 bp CDS (of the 621 bp) resulted in a protein corresponding to the first 202 amino acids (aa) (Figure S1A and S1B) of the ovine SCF (oSCF), in which the last 28 aa at the C-terminus was spanned into the putative primary proteolytic region of the long isoform i.e., s-SCF or (+) form and is identical to three of the GenBank oSCF protein sequences of 260 aa, 202 aa and 267 aa (Acc. No. AAB49491, CAA90620.1 and P79368), respectively.

### Rapid Amplification of cDNA Ends (RACE)

To obtain the full length cDNAs, we performed the 3′ and 5′ RACE experiments sequentially. Two different sets of primer (Table S2) were used for RT-PCR amplification in order to ascertain the corresponding 3′ and 5′ untranslated regions (UTRs) of the two different transcript variants i.e., (+) and (−).

### 3′ RACE – Detection of a Splice Variant of Ovine SCF

Initially, 3′ RACE cDNAs were prepared as described in materials and methods. One µl of this cDNA was used for the first round PCR amplification with the common CDS region forward primer and oligo(dT)_18_ modified as a reverse primer (Table S2). We got an unexpected short size of approximately 350 bp prominent amplicon since the expected 3′ UTR sequence with respect to other mammalian SCF mRNA species ranges from ∼500 bp to ∼4.5 kb. This was then gel purified, cloned into the TA cloning system and sequenced. To our surprise, the BLASTN sequence analyses revealed a 336 bp oSCF product (Figure 1B(b_5_)). Overlapping the 336 bp to the 621 bp CDS amplicon, we obtained a novel, truncated oSCF mRNA splice variant of 691 bp (without the 5′ UTR). Subsequent virtual translation of the ORF containing 546 bp resulted in a truncated oSCF protein of 181 amino acids with a unique C-terminus. The concomitant deletion in the shorter clone resulted in the substitution of aspartic acid (D) at aa pos. 175 with glutamic acid (G) i.e., D^175^G. Truncation would delete the C-terminal 93 aa residues of ovine s-SCF and fully conserved till G^175^ which is explained below. Henceforth, the new truncated protein isoform has a short stretch of 6 aa sequences right after the ‘G^175^’ residue ‘KTYKHS’ as its C-terminus (Figure S1B). This shorter form of oSCF has not been previously reported; however, short isoforms of SCF commonly known as membrane-bound form (m-SCF) corresponding to 245 aa lacking the proteolytic site have been reported as the (−) form of previously reported mammalian species including human [97], [100], mouse [11], cow [98] and avain [99]. The newly identified 181 aa oSCF (−) form differed from the 245 aa by the deletion of 64 aa at the C-terminus corresponding to the transmembrane and intracellular region. Hence, this novel cDNA variant could be recognized as the ‘membrane-anchored’ SCF protein (m-SCF) form and named as ‘SCF *truncated isoform-2*’, designated hereafter as (−) form since it lacks the primary proteolytic site. To our knowledge, this information of oSCF truncated (−) protein product is previously unreported in other mammal species especially in skin.

The remaining short 145 bp (after removing the adapter sequences from the oligo(dT)_18_ modified primer) including the polyA nucleotides belong to the 3′ UTR of ovine m-SCF (−) form. Mammalian genome scanning for the SCF gene represented that this novel 3′ UTR of ovine m-SCF (−) form corresponds to the intervening sequence in between exon 5 and exon 6 i.e., intron-5 of the (+) form. Here we hypothesis that the premature truncation could be the result of alternative use of the splice donor/acceptor site in the intervening sequences. Later, this short 3′ UTR amplification was confirmed (Figure 1B(b_5_)) in black and brown animals by direct sequencing but did not considered for further characterization such as SNPs.

In order to identify the 3′ UTR of the (+) form, we used the same 3′ RACE cDNA preparation as mentioned above. One µl was used for the first round amplification with the common CDS region forward primer and oligo(dT)_18_ modified as the reverse primer (Table S2). We obtained three different RT-PCR amplicons ranging from ∼700 to 1300 bp (Figure 1A(b)). At this stage it was difficult to substantiate this amplification. Hence, we performed three individual nested amplification sequentially using oligo(dT)_18_ modified as the reverse primer with the Nested forward primers (Table S2) for the consequent PCR reactions. All these amplified nested fragments were gel purified, cloned into the TA cloning system. Colonies were screened by colony PCR as well by restriction digestion, and the positive clones were subjected to sequencing.

Sequencing results showed three different sizes of fragment, one each from the Nested amplification (Table S2) viz. 597 bp (Figure 1B(b_3_)); 389 bp (Figure 1B(b_4_)); and 336 bp (Figure 1B(b_5_)) as positives for oSCF. Sequence analysis by BLASTN, BLASTP and ClustalW2 revealed all three products as ovine m-SCF (−) form and are identical to the one described above i.e., 336 bp for the reason that the (−) form override (+) form during the RT-PCR amplification. In other words, there exists a considerable difference in the mRNA expression level between these two transcript variants which is further explained in the later section. The rest of the amplicons were found to be non-specific including the two expected amplicons viz. ∼0.7/1.2 kb (Figure 1B(b_3_)) amplified from the primary RT-PCR amplification (Figure 1A(b) and B(b)).

**Figure 2:**
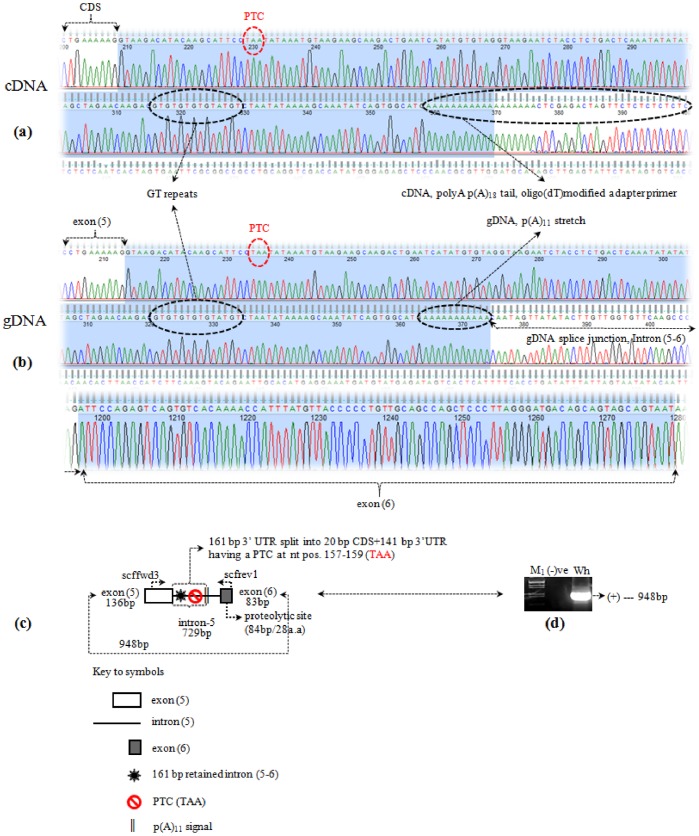
Sequencing chromatogram of cDNA in comparison with the gDNA amplification of oSCF gene. (**a**) Complementary DNA (cDNA) chromatogram shows the CDS, GT repeats and p(A)_18_ tail adapter primer as ‘black dotted oval mark’ and a premature termination codon (PTC) as ‘red dotted oval mark’ on the 3′ RACE product (336 bp, see Figure 1B(b_5_)); (**b**) Genomic DNA (gDNA) chromatogram shows the counter part of the above cDNA illustration (a) on exon 5 to exon 6 intervened by intron-5 sequences of the oSCF gene; (**c**) Amplification scheme of 948 bp splice junction covering exon(5)-intron-5-exon(6) of the oSCF gene with reference to human and mouse. The two exons 5, 6 are differentiated by ‘open and shaded box’ respectively. Arrows over the boxes indicate the fwd and rev primer. Different symbols on the intron-5 indicate the part of retained intronic sequences (161 bp) by a PTC along with the stretch of p(A)_11_ signal (see key to symbols below the diagram); (**d**) Gel picture shows the PCR amplification of 948 bp fragment corresponding to the above schema (c) of oSCF gene from blood gDNA; In the picture, arrow mark indicates the exact size of amplicon; **M_1_** indicates DNA size marker of λ-DNA EcoRI/HindIII digest; and (−)ve represents PCR negative control.

In all the above cases, we obtained always the (−) form, hence we designed a splice variant specific Nested forward primer (Table S2) with higher Tm for the (+) form. The primer was designed in between two exonic junctions (see Figure 1A and 2(c)) spanning into the proteolytic site viz. exon 5 into exon 6 in reference to the human, mouse, dog, horse SCF (source: Ensembl). The second round 3′ RACE amplification (Nested 1; see Table S2) was performed with 1 µl of the primary reaction product using (+) form specific forward primer (Table S2) and oligo(dT)_18_ modified reverse primer into a final PCR volume of 50 µl. The RT-PCR yielded an amplicon size of 855 bp (Figure 1A(b_1_)). Further third round amplification (Nested 2; see Table S2) yielded the expected 793 bp amplicon with some non-specific amplicons. The purified fragment of 793 bp (Figure 1A(b_2_)) was then cloned and sequenced. Sequence analyses by BLASTN and BLASTP confirmed the oSCF and named as ‘SCF *isoform-1*’, hereafter referred as (+) form, which is the counterpart of previously reported ‘soluble’ SCF (s-SCF) sequences in other vertebrate species [99]–[103] (source: GenBank, NCBI). Overlapping and editing of the 793 bp 3′ UTR fragment with the 621 bp CDS fragment, we obtained a total length of 1330 bp (without the 5′ UTR). The ORF of 825 bp corresponding to the deduced amino acid sequence of 274 aa revealed it as the s-SCF (+) form, indicating that this cDNA encodes the ‘soluble’ form of oSCF. This ovine s-SCF (+) form included the stretch of 28 aa recognized as a putative primary proteolytic site (Figure S1B) right after the D^175^ at its C-terminus as observed in the previously reported sequences [99]–[103]. The remaining long 505 bp (after removing the adapter sequences from the oligo(dT)_18_ modified primer) including the polyA nucleotides belong to the 3′ UTR of ovine s-SCF (+) form. The other two amplicons (data not shown) were found to be non-specific and omitted from further characterization.

### 5′ RACE

To determine the 5′ UTR of the oSCF (+) form, a gene specific 5′ RACE cDNA was synthesized using the proteolytic site specific reverse primer (Figure 1A; see Table S2) as described in materials and methods. Three µl of the dC-tailed cDNA was subjected to the first round RT-PCR amplification with the respective forward and reverse primer (Table S2). After the primary RT-PCR, the expected size of ∼780 bp amplicon was not detected on the gel. Consequently, a second round nested amplification was performed with a common CDS reverse primer and the forward adapter primer (Table S2) using 2–3 µl of the primary enriched RT-PCR reaction. Upon electrophoresis (1.5%), the secondary reaction yielded a single clear amplicon of ∼380 bp as expected. BLASTN results confirmed the sequenced clone of 364 bp (Figure 1A(c)) with the other mammalian s-SCF (+) form and was characterized as ovine s-SCF *isoform-1* i.e., (+) form. Overlapping and sequence comparison of this 5′ UTR clone to the 1330 bp (CDS +3′ RACE), resulted in a deduced 189 bp 5′ UTR sequence of ovine s-SCF (+).

The 5′ RACE RT-PCR for the oSCF (−) form was performed in a final PCR reaction volume of 50 µl containing 3 µl of the dC-tailed cDNA which was synthesised by a common CDS reverse primer (Figure 1B; see Table S2) along with all other necessary components as described in materials and methods. The first round enrichment PCR amplification was carried out using the same CDS reverse primer and the forward adapter primer (Table S2). The second round nested amplification was performed with another common CDS reverse primer and the forward adapter primer (Table S2) using 2–3 µl of the primary enriched RT-PCR reaction. Upon 1.5% gel electrophoresis, the secondary reaction yielded two distinct amplicons in the range of 200 to 330 bp. The two amplified 5′ RACE products were gel purified, cloned and sequenced. Sequence analysis revealed the two oSCF 5′ RACE products of sizes 325 bp and 215 bp (Figure 1B(d)). These two 5′ UTR products were not detected in the (+) form specific 5′ RACE cDNA (Figure 1A(c)) though the primer combination rely on the common CDS region. Hence, these two 5′ UTR products were characterized and named as ‘SCF *isoform-2a* (−) and *2b* (−)’, respectively (Figure S4(a_1_)). In order to confirm the amplification, this common 5′ RACE was repeated twice along with the (+) form specific 5′ RACE RT-PCR. Overlapping and sequence comparison of these two clones with the 691 bp (CDS +3′ RACE) revealed a deduced 144 bp, a 34 bp 5′ UTR sequences (after subtraction of the forward adapter primer sequence) for the two respective clones (325 bp, 215 bp).

### Genomic DNA – Spliceosomal Intron-5 Specific Amplification of oSCF

**Figure 3:**
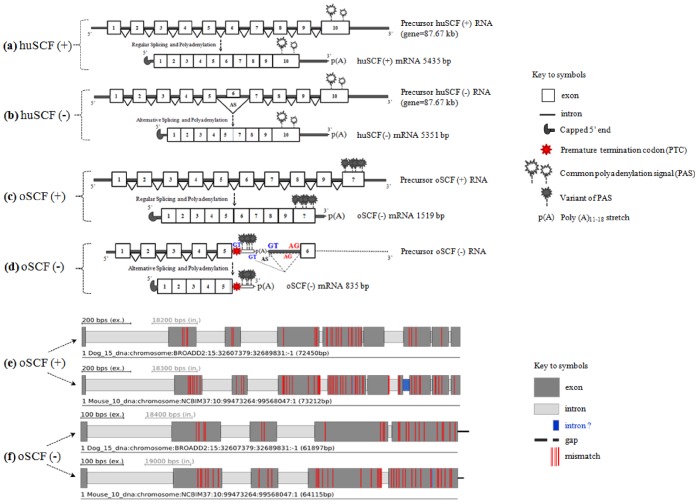
Gene architecture of ovine SCF gene in reference to human, mouse and dog. (**a**) Schematic representation of human SCF (huSCF) gene is shown. It consists of 10 exons (open boxes) intervened by 9 introns (linear black lines). Regular splicing and polyadenylation generates the full length huSCF mRNA *transcript variant-b*, a longer (+) form (5460 bp) encoding for a soluble product (273 aa; see Figure 5a); (**b**) The 84 bp exon 6 encoding for the 28 aa proteolytic site is skipped by an AS event of the huSCF gene is shown. The resultant full length huSCF mRNA *transcript variant-a*, known as (−) form (5376 bp) encodes for a membrane-bound product (245 aa; see Figure 5b); (**c**) Schematic representation of ovine SCF (oSCF) gene is shown. Regular splicing of exons 1–9/10(?) generate the full length oSCF (+) mRNA transcript (1519 bp) that encodes for a soluble product (274 aa; see Figure 5a); (**d**) Conversely, the possible AS events on intron-5 (Ref. human, mouse and dog) resulted in an alternative ORF with a premature termination (red symbol, PTC; see key to symbols). This resulted in retaining of 161 bp intronic sequence and completely eliminating (skipping of) the involvement of exon 6–9/10(?). The deduced protein sequence of this novel, shorter splice transcript variant (835 bp) resulted in 181 aa (see Figure 5b), a membrane-bound product of oSCF (−). In the above illustration, the open square or rectangle box symbolize exon and inverted triangle box symbolize intron. The open and shaded ‘black sparkle’ symbol on exon 10 (in 3a,b), exon ‘?’ (in 3c) and intron-5 (in 3d) all indicate the posssible position of predicted polyadenylation signal (PAS) sites. The two different sizes of the opened ‘black sparkle’ symbol (in 3a,b) denote the frequency of the common PAS such as ‘AAUAAA’ (8–12 times) and ‘AUUAAA’ (4–6 times) in the longer 3′ UTR of human, goat, mouse and rat. In contrast, the shaded ‘black sparkle’ symbol (in 3c,d) represents the other single basse ‘variants’ of PAS (see text in Results). Exon ‘?’ symbol (in 3c) represents the uncertainity of exon 10 position for oSCF (+). A ‘black hook’ symbolize the capped 5′ end and p(A) represents the polyA stretch on the preRNA, mRNA, respectively. The point of transcription termination (TAA) is symbolized as ‘red’ mark on intron-5 of oSCF (−) followed by the illustration of two possible mechanisms that resulted in a PTC of oSCF (−) (in dotted lines). The ASSP predicted constitutive and/or alternative splice donor (GT) and splice acceptor (AG) site(s) are labeled in blue and red letters respectively; (**e**) Schematic representation of the soluble oSCF (+) gene structure is shown. The exons/introns and the location of non-coding regions are determined in comparison to the mouse (chr 10) and dog (chr 15) SCF gene. The ‘intron (?)’ labeled in blue on the oSCF (+) in reference to mouse chr 10 indicates that the corresponding intron-7 is incomplete at that point i.e., it doesn’t show appropriate 5′ and/or 3′ splice sites; (**f**) Figure shows the gene stucture of membrane-bound oSCF (−). The ‘black line’ at the end of exon 5 of oSCF (−) in reference to mouse and dog indicate ‘gap’ i.e., coding sequence not found on the respective contig. The ‘vertical red lines’ over the exons indicate ‘mismatch’ of the oSCF (+) and (−) protein with dog (27 aa and 22 aa) and mouse (53 aa and 39 aa) SCF gene.

To verify the alternative splicing (AS) event that resulted in the shorter mRNA transcript i.e., ovine m-SCF (−) form, we amplified the intervening sequence between two exons. The sequenced chromatogram from the cDNA and gDNA of oSCF illustrating a PTC followed by the p(A)_11/18_ tail signal is shown in Figure 2(a,b), respectively. The reference SCF genomic locus at the exon 5-intron(5)-exon 6 splice junction was determined in comparison to the orthologous SCF gene assembly of human, mouse, rat, cow, horse and dog (source: Ensembl). The genomic DNA (gDNA) was obtained from the blood of white merino sheep. A expected amplicon size of 948 bp amplicon (Figure 2(d)) was amplified using an exon-5 (common CDS) specific forward primer and exon 6 specific reverse primer (+ form, proteolytic site; Table S2) as shown in Figure 2(c). Sequence analyses and orthologous comparison of the oSCF gene product (948 bp) with other mammals revealed that the first 136 bp corresponds to exon 5, followed by an intron-5 of 729 bp (Figure S4(b)) and an exon 6 containing 83 bp which encodes for the primary proteolytic site. This result was compared with the shorter cDNA transcript. The first 161 nt including a 11 bp polyA (pA) stretch of the intron-5 exhibited 100% identity to the nt pos. 668–835 of the shorter cDNA (Figure S4(c)). However, careful annotation of the 161 nt unveil a premature stop codon at nt pos. 21–23 of the 729 bp intronic sequnce. Figure 3 shows the oSCF gene structure(s) in reference to mouse, dog and human SCF gene (see also Figure S2 for the humanSCF alternative forms). The overall similarity for this 948 bp DNA splice region in other vertebrates was found to be highest with goat and cow SCF (99 and 94%) where as the lowest was detected with chicken and zebra finch SCF (62%).

Intron-5 has a constitutive 5′ splice donor (GT) at its start and six other alternative isoform/cryptic splice donor (GT) sites (Figure S4(b)). Similarly, it has a constitutive 3′ splice acceptor (AG) site exactly at the end of the intron-5 and five other alternative isoform/cryptic splice acceptor (AG) sites (Figure S4(b); see also Figure 3(d)) as predicted by ASSP, RegRNA [91], [92]. Seven important sequences, the so called the ‘branch site’ (BS; Figure S4(b)) viz. CU(Pu)A(Py) are located 20 to 75 bases upstream of the predicted acceptor site. Of which ‘CUGAC’, ‘CUAAU’ and ‘CUGAU’ are considered at most to be the main branch point sites that could be involved in the AS event. PolyADQ [88] prediction revealed two polyadenylation signal (PAS) of the type ‘AAUAAA’ in the 729 bp gDNA (intron-5; Figure S4(b)), but present after p(A)_11_ stretch hence was not considered to be part of the polyadenylation. However, here we hypothesis that the two other single base variants of ‘AAUAAA’ [104] such as type ‘UAUAAA’ at nt pos. 24 (right after the stop ‘TAA’), 126 and ‘AAUAUA’ at nt pos. 83, 124 found just before p(A)_11_ bp stretch could be responsible for the polyadenylation process of the shorter mRNA (−) transcript (Figure S4(b); see also Figure 3(d)). These two strong polyA signals are also present in the cDNAs of the respective oSCF mRNA transcripts (Figure S4(c)). The other two single base variants ‘AAUAGA’ and ‘UAUAAA’ detected at 407 nt and 427 nt (Figure S4(b)) away from (pA)_11_ stretch, respectively, in the same intron, are not considered further the AS analyses.

### Chromosome Location and Genomic Structure of the oSCF (KITLG)

**Figure 4:**
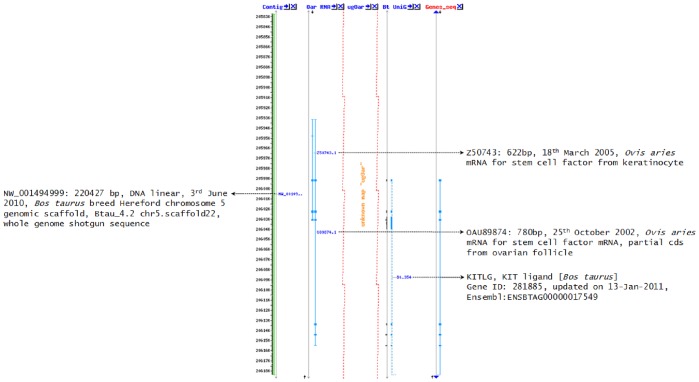
NCBI Map Viewer of ovine SCF (oSCF) aligned to the bovine SCF gene. The comparative map depicts unknown map region (in red dots) of the oSCF gene to the counter-part of bovine SCF (KITLG) on chr_5:Btau_5.2 displaying regions of 20,582,700–20,618,400 bp. Arrows indicate mapped GenBank records (Acc. No.) with description, respectively.

Upon scanning through the sheep genome Oarv2.0 (March 2011 – till date) covering position from 124,495,129 to 124,515,933 of Ovine (Texel) Version 2.0 (current) Genome Assembly we obtained the mere size of OAR3: 20.8 kbp (data not shown). It represents only 19% of the known SCF gene size when compared to human (108.74 kbp), mouse (104.78 kbp), cow (122.28 kbp) and dog (100.21 kbp) (source: Ensembl). The gene encoding the ovine SCF (NCBI gene ID: 443371) is located within a syntenic group on chromosome 3 [105], corresponding to the *Sl* or *kitlg* gene locus. This portion of ovine chr 3 is homologous to cattle chr 5. Hence, a comparative chromosomal mapping (Figure 4) was performed at the NCBI Map Viewer [84] of the sheep SCF to the cow SCF i.e., *O.ari* chr 5 to *B.tau* chr 5 and *O.ari* chr 3 to *B.tau* chr 5. Genomic DNA and cDNA sequence comparison and prediction [63] revealed that the oSCF gene consists of 9 exons interrupted by 8 introns to the dog (Figure 3(e)), pig, horse SCF gene where as in comparison to human, chimpanzee, marmoset, mouse (Figure 3(e)) and rat including the unfinished alpaca genome (source: Ensembl), oSCF gene has been characterized by 10 exons and 9 introns. Comparative analyses of oSCF (+) protein to the dog and mouse SCF gene assembly exhibited 96/90.1 and 93/80.6 match ratio and % identity, respectively. Similarly, oSCF (−) protein showed the match ratio and % identity of 91/87.2 and 90/77.7 to dog and mouse SCF gene assembly, respectively. Among the 9/10 exons, it is predicted by gene annotation (source: Ensembl) that the exon 5 and exon 6 has its importance in determining the final protein product through AS event(s) and the longer exon 10 (9) corresponds to ∼4.4 kb 3′ UTR in human, chimpanzee, mouse, rat and goat in contrast to the shorter 3′ UTR in sheep (reported in this study), cow, pig, horse, dog, cat and panda (source: Ensembl).

### Protein Characterization of the Ovine SCF s-SCF (+) and m-SCF (−)

The molecular mass of the oSCF isofoms presented in this study as predicted by EditSeq, DNASTAR [62] is 31.1 kDa and its theoretical iso-electric point is 5.236 for the s-SCF (+) isoform corresponding to the 274 aa. Similarly, the m-SCF (−) isoform has a molecular weight of 20.6 kDa with a theoretical iso-electric point of 6.002 for the 181 aa residues.

**Figure 5:**
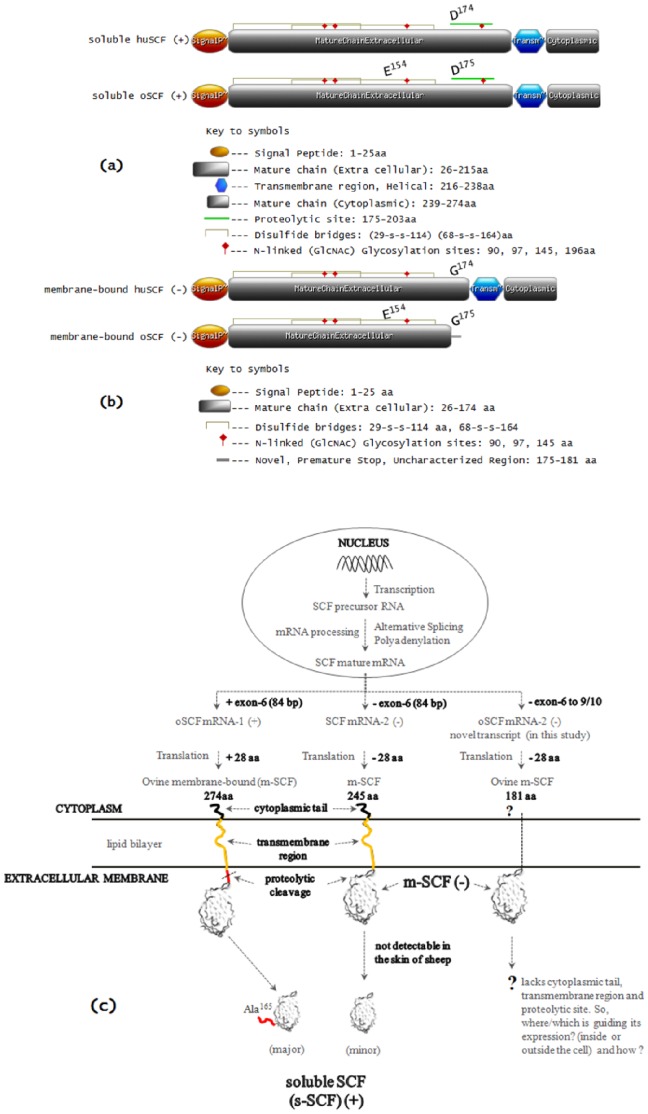
Schematic representation of the topological characteristics of two different ovine SCF (oSCF) protein products in comparison to human SCF (huSCF). (**a**) Illustrates the identical topological features for the soluble oSCF (+) and huSCF (+) which corresponds to the 273 aa *vs.* 274 aa, respectively. The D^174/175^G represents the change of aa residue for the alternative natural variant i.e., right at the proteolytic site (28 aa, ‘green line’). The difference in the position is due to sequence divergence of soluble oSCF (+) which has an additional ‘Glu’ residue at ‘E^154^’ (see Figure S3d). (**b**) Demonstrates the difference in topological features of the membrane-bound oSCF (−) and huSCF (−) which corresponds to the 181 aa *vs.* 245 aa, respectively. This novel ovine m-SCF (−) has a unique C-terminus with an additional uncharacterized 6 aa residue (176–181, see key to symbols) right after D^175^G. Given below the diagram (in 5a, b) are the appropriate topological features (see key to symbols) of human and ovine soluble SCF (+) and membrane-bound SCF (−) with referencce to UniProt ID. P79368 and P21583; (**c**) Schematic representation of ovine SCF gene transcription and translation in skin (hypothetical view). The corresponding oSCF protein products, s-SCF (+) and m-SCF (−) and their topological characteristics are labeled and highlighted respectively.

Topological features of both the isoforms (+ and −) of ovine SCF in comparison to the human SCF is given in Figure 5(a,b). In ovine s-SCF (+) form, the first 25 amino acids contain features (Figure 5a) of a signal peptide, followed by an extracellular mature chain (aa pos. 26–215), a putative hydrophobic transmembrane region (aa pos. 216–238), and a 35 amino acid intracellular domain (aa pos. 239–274). The 28 aa proteolytic site resides at aa pos. 175–202 which includes a N-linked glycosylation site at aa pos. 196. The four cysteine residues found within the extracellular domain that may result in disulfide bridges viz. (29-s-s-114), (68-s-s-164) and the three other N-glycosylation sites found in the extracellular domain at aa pos. 90, 97, 145 are conserved with all the mammalian s-SCF (Figure S3(d)). The above described features of ovine m-SCF (−) form has been shown in Figure 5(b), which depicts the shortage/deletion of the primary proteolytic site including an N-glycosylation site, a trasmembrane domain (necessary to make a soluble product) and a cytoplasmic domain. The sketch of oSCF gene transcription and translation is shown in Figure 5(c).

### Conservation of the Ovine s-SCF Protein Isoforms

**Figure 6:**
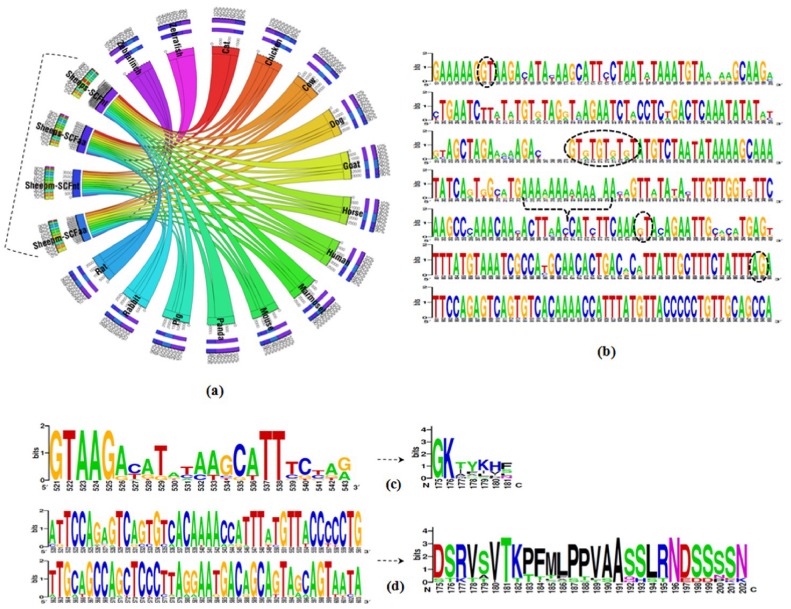
Graphical representation of evolutionary conservation of sheep SCF isoforms. (**a**) Percent of conservation was calculated for sheep, goat, cow, pig, cat, dog, panda, horse, human, chimpanzee, marmoset, mouse, rat, rabbit, chicken, zebra finch and fishes, such as zebra fish, gold fish using the multiple sequence alignment (MSA) tool, ClustalW2 with four different datasets (provided on request). The Circos graphical table view represents the sheep soluble, s-SCF (+) and membrane-bound SCF, m-SCF (−) nucleotide (nt) and protein (aa) as the query sequences (in black dotted left bracket) against 17 other vertebrate species. Four different colour small bars on the query sequences represnts the four different data sets of sheep s-SCF (+) and m-SCF (−) nt/aa sequences. The 15 different colour ribbons passing through each other represent respective vertebrate species and the percent identity is indicated outside as the boundary. The four different colour small bars over the 15 vertebrate species as against 15 different colour small bars above the sheep query sequences represnts the percent identity among each other. The scale over each species (above small bar) represents the total score obtained from the sequence coverage; (**b**) Graphical logo representing the conservation of oSCF splice junction (intron-5) which was generated by MUSCLE alignment (manually predicted for other species), depicting the GT repeats (black oval dotted lines) proximal to the poly(A)_11_ stretch (black dotted right brace symbol). The constitutive splice donor (GT) and acceptor (AG) sites are circled by black dotted lines along with one of the proposed usage of alternative/cryptic splice donor site (GT) (see Figure 3f). Numbers below the logo indicate the nucleotide/amino acid position of the MUSCLE aligned sequences; (**c**) Logo representing the 23 nt conservation of the m-SCF (−) form (novel sequence reported in this study) and its deduced 7 aa new C-terminus is shown; (**d**) Graphical logo representing the 84 nt conservation of the s-SCF (+) form and its deduced 28 aa proteolytic site is shown. Numbers below the graphical representation of (c), (d) indicate the actual nucleotide/amino acid position. The height of the letters on each logo represents the relative frequency of each nucleotide/amino acid in a given position.

Using the default settings of NCBI, BLASTN and BLASTP search was conducted with ovine s-SCF (+) form of 825 bp CDS and its deduced 274 aa as query sequences, respectively. Multiple sequence alignments (MSA) [64] (Figure S3(a,b,d)) of the nucleotide and the deduced amino acid sequences belonging to different mammalian representatives indicated that the sheep SCF was highly conserved and found to have between 57% and 99% nucleotide similarity and 19% to 99% protein identity (Figure 6(a,d)). The highest identity was with the goat SCF where as the lowest was with gold fish and zebra fish SCF for nucleotide and protein respectively viz. goat (99/99%), cow (97/98%), pig (95/94%), cat (94/90%), panda (93/90%), horse (93/89%), dog (92/88%), human and chimpanzee (91/86%), rabbit (90/84%), marmoset (90/83%), rat (88/82%), mouse (87/80%), zebra finch (74/55%), chicken (73/53%), zebra fish(59/19%) and gold fish (57/26%). The graphical logo representing the conservation of oSCF splice junction (intron-5) with GT repeats, poly(A)_11_ stretch and the constitutive splice donor (GT) and acceptor (AG) sites are shown in Figure 6(b).

Similarly, the deduced 181 aa sequence from 546 bp CDS of the ovine m-SCF (−) form shares 49–99% identity with the predicted m-SCF (−) form of the same length of a number of other vertebrate species (Figure 6(a,c); see also Figure S3(a,c)) including avian SCF. The highest identity was with the goat (99%) followed by cow (95%) where as the lowest was noticed with chicken (49%) followed by zebra finch SCF (51%).

### Skin Expression of the Two Ovine SCF Splice Variants

**Figure 7:**
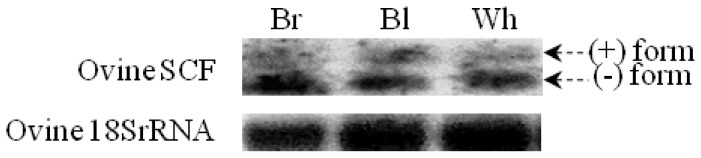
Expression of ovine SCF in skin. Northern blot analysis show ovine SCF (+) and (−) mRNA expression. Ovine 18S rRNA was used as an internal control. Northern blot analysis was carried out with a DIG-labeled cDNA probe for SCF and 18S rRNA (see Table S2) as described in Materials and Methods section. Br, Bl, Wh represents individual of Brown, Black and White merino sheep, respectively.

Initially, to verify any eventual difference(s) between the expression level of two different splice variants of oSCF (+/−) four sets of primer (summarized in Table S2) were used as described in materials and methods. Three individuals of white, black and brown animals were subjected to a single round RT-PCR amplification. The RT-PCR reactions gave fragments (see Table S2 for details) exhibiting almost the same level of band intensity for both the (+) and (−) form (data not shown). In contrast, Northern blot analysis showed substantial differences in the expression oSCF between (+) and (−) form (Figure 7). At this juncture, we propose that the oSCF gene expression in white, black and brown animals at mRNA transcript level is mediated via an intron-5 AS event (Figure 3(c,d). However, both forms (+/−) are biologically active and reported to have different effects on cells [9]–[11], [20]. The regulation of processing of the proposed secondary proteolytic cleavage site encoded by exon 7, could play a critical role in the function of membrane-associated SCF (−) protein [10].

### SCF UTR Regulatory Motifs that Affect mRNA Stability

The different 5′ and 3′ UTR sequences of sheep SCF(s) were searched against the UTRdbases [86], [92] for the post-transcriptional associated regulatory elements located in the 5′ and 3′ untranslated regions. Among the *cis*-elements that play a role in translation down-regulation are an upstream open reading frames (uORFs) [106] at nt pos. 80, 193 for the (+) form and at nt pos. 35, 148 for the (−) form; and a polypyrimidine motif, known as terminal oligopyrimidine tract (TOP) [107] at nt pos. 1, 5 located in the 5′ UTR of oSCF (+) form are shown in Figure S4(a_1_). The critical regulatory sequences, known as Cytoplasmic Polyadenylation Elements (CPEs), are AU-rich elements (AREs) [108] located in the 3′ UTR near by the canonical nuclear polyadenylation element (AAUAAA), key sequence features controlling mRNA deadenylation and decay. Surprisingly, sheep SCF mRNA has the following single base variant [104] of the type CAUAAA (nt. 1076), AAUGAA (nt. 1080), UUUAAA (nt. 1091), UAUAAA (nt. 1225), AAUAGA (nt. 1441), and AACAAA (nt. 1095, 1183, 1486) for the (+) form (Figure S4(d)) and UAUAAA (nt. 691, 792), AAUAUA (nt. 750, 791) and AGCAAA (nt. 799) for the (−) form (Figure S4(c); see also Figure S4(b)) as its PAS, which are required for proper poly(A) addition. The other regulatory 3′ UTRs found in the oSCF (+) form are Bearded (BRD) Box [109], ‘AGCTTTA’ at nt pos. 1088, 1391 and Musashi binding element (MBE) [110], ‘ATAGT’ at nt. pos. 1422, 1458 (Figure S4(d)).

### mRNA Structural Characterization

**Figure 8:**
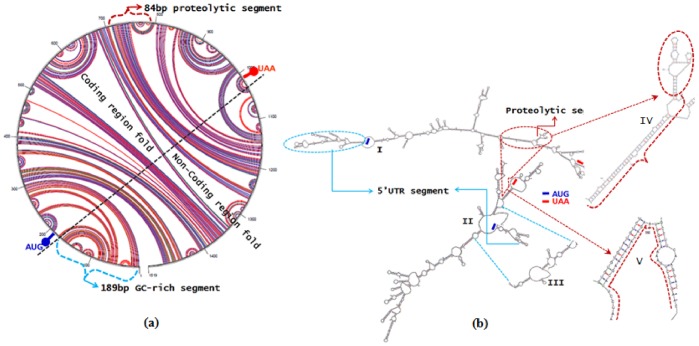
Ovine SCF RNA fold model explaining the observed results. (**a**) Secondary structure predicted for the major 1519 nt s-SCF (+) mRNA. The m-fold circle diagram, generated by minimal free energy (dG = −437.3 kcal/mol) indicate G–C, A–U and G–U base pairs in red, blue and green arc lines, respectively. It also differentiates the 825 nt coding and 505 nt non-coding region fold by the vertical black dotted line which divides the circle diagram. The 189 nt GC-rich segment of 5′ UTR which forms a dense secondary structure and the presumptive splice site of 84 nt proteolytic segment are highlighted and labelled in cyan and dark red dotted line respectively. The start (ATG) and stop (UAA) codons are labeled in bold blue and red letters respectively. The numbers present outside the circle diagram indicates the nucleotide position in base pairs (bp) at every 100 bp intervals; (**b**) Stemloop secondary structure representation of 1519 nt s-SCF *isoform-1* (+), 825 nt m-SCF *isoform-2a* (−) and the partial image depicting the 5′ UTR segment fold of 725 nt m-SCF *isoform-2b* (−). The major structural features in illustration (a) and (b) are labeled alike. Except the GC-rich 5′ UTR segments where in I (189 nt), II (144 nt) and III (34 nt) represent the difference in 5′ UTR fold (light blue/cyan dotted arrows pointing to the corresponding dotted oval shape; see also Figure S4(a_1,2,3_). Similarly, IV (+84 nt) and V (−84 nt) represents potential fold difference for the proteolytic segments (dotted dark red arrows directd to the corresponding expanded structures).

In addition to the coding region (+/−84 bp proteolytic site), SCF mRNA(s) has four notable features relevant to its secondary structure (Figure 8(a,b)). First, the 5′ UTR is enriched in G+C nucleotides (Figure S4(a_3_)), with 64% and 60% or 56% (G+C content) in the 189 nt and 144 nt segment for the (+) and (−) form respectively. Second, the 5′ UTR segment has specific trinucleotide elements (Py-G-C; Figure S4(a_1_)), in our case ‘CGC’ at nt pos. 4, 16/18, 33, 36, 68, 100, 154, 176 and and ‘TGC’ at nt pos. 66, 81, 94, 103, 109, 116, 146, 157, 179 that are known to cause DNA polymerase pausing [111]. These trinucleotides (CGC, TGC) which accounts for 9.6% of the ovine s-SCF (+) 5′ UTR segment (189 bp), could attribute to the smaller 5′ RACE cDNA product(s) for example, the one of oSCF *isoform-2a* (−) (Acc. No. GU386374; Figure S4(a_1_)). Third, sheep SCF mRNA contains a frequent hexamer direct repeats (DRs) i.e., ‘CGCTGC’ (1.6%) at nt pos. 100, 154, 176 located in the 5′ UTR of (+) form (also present in (−) form but nt pos. differs; see Figure S4(a_1_)). This repeat is highly conserved among mammalian SCF mRNAs (Figure S4(a_2_)). Fourth, the 3′ UTR has a DRs containing a consecutive hepatamer ‘GTGGGGG’ at nt pos. 1461, 1468 in the (+) form which is highly conserved only in goat (Figure S4(d)). In contrast, a perfect dinucleotide repeats (GT)_5_ at nt pos. 775 is present in between the hepatamer tandem repeats ‘CAAATAT’ at nt pos. 748, 801 in the (−) form, are also highly conserved with goat but varies only in the dinucleotide repeats with ‘AT’ for cow, dog and horse as shown in Figure S4(c). In between this feature, there exists a putative alternative isoform/cryptic splice donor (GT) at nt pos. 117 of 729 bp intron-5 of oSCF (Figure S4(b)) with 92.5% score as predicted by the ASSP [91] classification.

### MicroRNA Targets: Another Type of *cis*-acting Regulatory Element

The above described differences between the two ovine splice variants i.e., (+) and (−) in the conservation of non-coding sequences (Figure S4(d)) suggests that the 3′ UTRs, might have a functional role in gene regulation.

**Figure 9:**
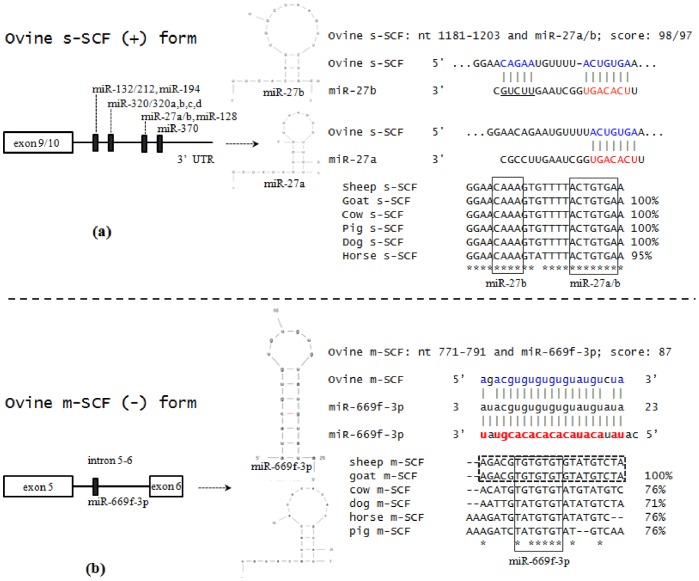
Location of potential miRNA target sites on the 3′ UTR sequences of oSCF (+/−) . Vertical black bars on the schematic diagram represent miRNA target sites on the 3′ UTR region. Open and dotted boxes represent potential miRNA target sites and sequence conservation, through evolution in sheep, goat, cow, dog, horse and pig. (**a**) The predicted potential binding site of miR-27a,b on the 3′ UTR of ovine s-SCF (+) and stemloop structure (mfold) of the miR-27a,b is shown. The seed sequences (nt. 2 to nt. 8) of the miR-27a,b is shown in red, bold letters. The target nucleotides involved in pairing are shown in blue, bold on the ovine s-SCF (+). The nucleotides involved in pairing outside the seed sequence are underlined in black; (**b**) The predicted potential binding site of miR-669f-3p on the 3′ UTR of ovine m-SCF (−) and stemloop structure (mfold) of the mature miR-669f-3p is shown. The miR-669f-3p target sequence is located on the non-coding intron-5 closest to exon 5. The mature miR-669f-3p is shown in red, bold letters. The target nucleotides involved in pairing are shown in blue, bold on the ovine m-SCF (−).

A number of potential miRNA target sites are found within the longer ∼4.4 kb 3′ UTR sequence of human SCF (data not shown). However, in sheep, the analyzed miRNA sites that are located in the 505 bp 3′ UTR of the ovine s-SCF (+) form belongs to the miRNA families of miR-27a/b, miR-194, miR-128, miR-370, and two sites for miR-132/212, miR-320/320abcd (Figure 9(a)) where as miR-669f/a/o-3p, miR-466b and miR828b are detected on the shorter 3′ UTR segment (144 bp) of ovine m-SCF (−) form (Figure 9(b)). Interestingly, the 8-mer miRNA (miR-669f) has a high context score (87 percentile) which binds to the 21 nt off 23 nt of the 3′ UTR target of the oSCF (−) form (Figure 9(b)).

### Homology Modeling

The predicted three-dimensional structures of the deduced SCF protein corresponding to 141 aa and 132 aa residues were modelled using the best matched PDB templates with 90–100% identity to the individual chains such as 1EXZ, 2E9W:chainC,D and 1SCF. The structure was predicted as using Modeller 9v2 [54] as described in materials and methods. The quality assessment of the modelled structures were performed at SWISSMODEL Workspace [52].

Topologically, the modelled oSCF structure has a core of four alpha(α)-helices (αA, αB, αC and αD) and two antiparallel beta(β)-strands arranged to form a protomer i.e., β1 between αA and αB and β2 between αC and αD. Apart from this, it consist of three other additional unique conformations i.e., one-turn helix, αB’ between β1 and αB, an hairpin loop between αB and αC at the dimer interface, and an extra one-turn helix, αD’, in the C-terminal extension [112]. This conformation is in accordance with the crystal structure determined for 1EXZ, 1SCF and 2E9W [112]–[114]. The best models were choosen based on the quality assessment reports of ProCheck [115] and Promotif [116]. The calculated Ramachandran plot showed 91–95% of the aa residues lie in the core region for those structures modelled using Modeller 9v2, representing the most favourable combinations of phi-psi values, guiding to the better stereochemical quality of the oSCF protomers while for the one modelled using an automated comparative protein modeling server at SWISS-MODEL, exhibited 70.7% in the core region. Six out of eighteen modelled strcutres were picked and the superimposition of one of the oSCF monomer model to the PDB template 1EXZ:chainB is shown in Figure S5. All these observations suggest correct structure and folding for the modelled putative oSCF.

### Molecular Phylogenetic Analyses

The evolutionary divergence of sheep SCF cDNAs and its corresponding protein sequences were studied using other vertebrate sequences from the GenBank, Ensembl and necessary BLAT searches. Except the s-SCF (+) form, the spliceosomal intron junction on the DNA sequences and the m-SCF (−) form were predicted manually in accordance to the ovine SCF sequences.

Five different alignments were constructed for the phylogenetic analysis (data sets provided on request): 1) SCF (+) CDS nucleotide data sets (14 mammals, 2 avian and 2 fish, 822 nt unambiguously aligned characters); and 2) SCF (+) CDS deduced protein sequences (13 mammals, 2 avian and 2 fish, 274 aa unambiguously aligned characters); 3) Predicted SCF (−) CDS nucleotide data sets (12 mammals and 2 avian, 543 nt unambiguously aligned characters) and 4) predicted SCF (−) CDS deduced protein sequences (11 mammals and 2 avian, 181 aa unambiguously aligned characters); (5) Predicted SCF DNA sequences concatenated to the exon 5-Intron(5)-exon 6 (12 mammals and 2 avian, 948 nt unambiguously aligned characters). Unambiguously MUSCLE [47] aligned sequences were confirmed by eye, and unnecessary gaps were excluded from the alignments with GBLOCK program [65] prior to phylogenetic analyses. Phylogenetic relationships were inferred from all five alignments using neighbour-joining (NJ), maximum likelihood (ML) and Bayesian inference (BI) methods as described in materials and methods. The best fit models were scrutinized from 88 nt models [81] and 56 aa models [82] based on the AIC/AICc/BIC/−lnL scores. After the appropriate model selection, the final trees were constructed using the simple p-distance for NJ method, JTT+G, a protein model for ML, BI methods and GTR+G and/or HKY+G for ML, BI as the nucleotide substitutions models. Numbers on the respective nodes denote the supportive bootstrap values of NJ, ML in percentages, and Bayesian posterior probabilities, respectively with the separation of a solidus (/) symbol (Figure S6(a-e)). Apart from the regular GTR+G, HKY+G models, the other useful nucleotide substitution models for our evaluated data sets include TIM3+G, TPM3uf+G, TVM+G, TrN+G and TPM1uf+I+G. All these evaluated models differ in their respective scores by ±5 and produced consistent tree topologies.

All five constructed phylogenetic tree (Figure S6(a-e)), based on oSCF nucleotide and protein sequences (5 different data sets, provided on request) produced similar monophyletic clusters as mammals, avian, and fishes indicating that all the species delineated successfully and was found to be in harmony with the established positioning of these vertebrates. In the tree (Figure S6(c,d,e)) pig-1, pig-2 represents two possible predicted m-SCF amino acid, nucleotide and DNA splice junction sequences, respectively (data sets provided upon request). Note: The s-SCF and m-SCF protein sequence of chimpanzee has 100% identity, hence omitted from the MLA and further tree analyses.

## Discussion

Stem cell factor (SCF), characterized as mast cell growth factor (MGF), is a multifunctional growth factor for haematopoietic progenitors, germ cells, melanocytes and mast cells [117]. It is mainly produced by fibroblasts, keratinocytes, endothelial, bone marrow, thymic stromal and small cell lung cancer cells [117]. Moreover, SCF mRNAs (cDNAs) structure and expression have been identified in a variety of other tissues such as brain, kidney, lung, and placenta (source: Ensembl, Aceview). Perhaps one of the more interesting improvements in the area of hair follicle melanogenesis is the isolation of SCF. Although considerable information on SCF cDNA sequences are available in the GenBank repository (NCBI) for several mammal species, the full-length mRNA (cDNA) structure for sheep (*Ovis aries*) remains unclear untill now (source: Oarv2.0, GenBank, NCBI, March 2012). To our knowledge, there is no experimental evidence or report for the existence of ovine SCF in skin. Taking into account the potential role exerted by SCF in hair follicle melanogenesis [16], [36], ovine SCF cDNAs were amplified, cloned and sequenced from the skin of white merino sheep (Figure 1 and 2). Nucleotide sequence analyses and the deduced amino acid sequences disclose the orthology of ovine SCF gene with other mammal species (Figure 3 and 6(a); see also Figure S3(a-d)). Herein, we report for the first time, the isolation of the two alternatively spliced, full-length oSCF mRNA (cDNA) transcripts such as the longer, SCF *isoform-1* (+) widely known as ‘soluble or secreted’ (s-SCF) form and a shorter, SCF *truncated isoform-2a/b* (−) (*a/b* denotes the 5′ UTR differences; see Figure S4(a_1_)) possibly characterized as the ‘membrane-anchored’ (m-SCF) form from the skin biopsies of white merino sheep. In which, the later has been identified and characterized as ‘novel’ in that the truncated (−) form reported in this study is devoid of 28 aa proteolytic site including a N-linked (GlcNAc) Glycosylation sites and the 23 aa transmembrane region followed by the cytoplasmic tail corresponding to 35 aa in comparison to the commonly known SCF (+) form (Figure 5(a,b)). As a result of the premature termination codon (PTC) in intron-5, the novel protein isoform has a unique, truncated, short stretch containing ‘KTYKHS’ (6 aa) as its novel C-terminus (Figure 6(c,d) and Figure S3(b,c); see also Figure S1B). It has been proposed that soluble SCF is derived from the transmembrane form by proteolytic cleavage within its extracellular domain [25].

The primary oSCF cDNA fragment (621 bp; Figure S1A,B) corresponding to the CDS of 606 bp reported here closely matches to the previously described oSCF sequences [118], [119]. The only exception in the deduced 202 aa is at Q^134^ (glutamine) which has been reported as E^134^ (glutamic acid) [118]. However, it has been confirmed as Q^134^ in our virtual translation from the sequenced oSCF cDNA sequences (in this study) and is in agreement with the previously reported oSCF [119]. Besides, the bovine SCF amino acid sequence also has a Q at pos. 134 [98] (Figure S3(d)). In the present study, the coding region of the longer, oSCF (+) form is identical to that of previously isolated human SCF (Figure 5(a)) and corresponds to the other mammalian counterpart of SCF (+) form (Figure S6(a,b)). In contrast, the shorter oSCF (−) form identified in the present study, has a premature termination codon (PTC) at intron-5 (Figure S4(b)) leading to the complete skipping of exon 6 to exon 9/10 viz. differing in the cassette exon (CE 6–9/10). Since this splicing event leads to complete elimination of the proteolytic site, a transmembrane region and the subsequent cytoplasmic domain of the oSCF (+) protein (Figure 5(a,b)), the resultant product of the shorter isoform would not be secreted (Figure 5(c)). Perhaps, the cell would require an alternative mechanism for producing this shorter isoform. At this stage, it is important to examine which cell type (melanocyte, keratinocyte and fibroblast) is producing this truncated oSCF (−) form and where it is expressed either intracellular or extracellular environment (Figure 5(c)) will elucidate the functional and biological significance of this oSCF (−) product in hair follicle melanogenesis. In comparison to the previously reported 245 aa membrane-bound isoforms in other mammals [5], [6], [25], [136], it is possible that the SCF encoded by this shorter ovine cDNA would remain membrane-bound as it lacks the necessary primary proteolytic cleavage site to produce a soluble form [25]. This form of SCF mRNA thus can produce only membrane-anchored SCF [6], [25], [120]. While the *in vivo* roles of soluble versus membrane-bound SCF are unclear, like other membrane-associated growth factors (e.g. Transforming growth factor, TGF-α and Tumour necrosis factor, TNF), is thought to be involved in intercellular communication [10].

Over all sequencing results revealed that the SCF gene like in other mammal species, oSCF primary transcripts also undergo alternative splicing (Figure 3(c,d)) with the exon-intron boundary location, size and amino acid composition of the alternatively spliced region being highly conserved [5], [6], [97], [98]. Alternate splicing of intron-5 (CE 6–9/10; skipping of exons 6–9/10) of SCF might therefore provide a mechanism by which the specific type of cell (melanocyte, keratinocyte and fibroblast) could regulate the relative amounts of soluble and membrane-bound SCF that were produced inside the cell (Figure 5(c)). In addition, to the known variant lacking exon 6, an alternative splicing of exon 4, resulting in four possible isoforms was reported in pig [121]. Analysis of oSCF to human, mouse, rat and dog genomic clones showed identical exon/intron boundaries of the oSCF gene architecture (Figure 3(a,b,e,f); see also Figure S2). While performing oSCF cDNA amplification including 5′ and 3′ RACE, we have analyzed a number of independent oSCF clones and have found no evidence for an alternatively spliced form encoding a membrane-anchored isoform corresponding to the 245 aa as reported in other vertebrate species [5], [6], [100], [122]. From our RT-PCR results, it seems that this particular mRNA species is completely absent in sheep atleast in skin. In other words, the spliceosomal machinery in the skin of sheep failed to generate the oSCF mRNA (−) form which encode for the 245 aa. Instead, it generates the above described truncated shorter ovine m-SCF (−) form (Figure 3(d)). Henceforth, we assume that spliceosomal machinery eliminates the probability of SCF mRNA(s) processing in a similar manner across species i.e., the retention of exon 7 to exon 9/10 in the (−) form (245 aa) (compare Figure 3(b,d)) which has been reported in several studies [5], [6], [100], [122].

The original descriptions of the cloning of SCF including location of introns in the coding regions have been reported for human and rat SCF genes [100]. In comparison to other vertebrate species, the SCF gene is composed of at least 9/10 exons (Figure 3; see also Figure S2) ranging from ∼63 bp to <4 kb in length which are intervened with a wide range of varying length of 8/9 introns viz. ∼700 bp to <34 kb (source: Ensembl). The locations of introns in the coding region of SCF are conserved in rats, mice, and humans [5], [100]. The total length of SCF gene ranges between ∼72 kb to ∼87 kb (source: Ensembl). Previous reports on oSCF Northern blot analysis revealed a major SCF mRNA transcript of ∼6 to 6.5 kb in ovarian follicles, corpus luteum and stroma [118], [119]. In other species, a major band between 5.5 and 6.5 kb has been described in human [100], mouse [5], [11], cow [98], pig [101] and chicken [103]. Shorter and less abundant SCF mRNA species have been reported in the mouse [5] and the chicken [103] (source: Ensembl). From our Northern blot ananlysis and long range 3′ RACE RT-PCR, it seems that the larger SCF transcript (∼6 kb) is not expressed in ovine skin.

The human and mouse SCF gene on AceView program [89] revealed 18 different ‘GT-AG’ introns and the transcription produces 8 different mRNAs (Figure S2 and Table S3A), 7 alternatively spliced variants and 1 unspliced form. There exist 2 probable alternative promotors, 2 non overlapping alternative last exons and 5 validated alternative polyadenylation sites (Table S3C). The mRNAs appear to differ by truncation of the 5′ end, truncation of the 3′ end, presence or absence of 9 cassette exons, overlapping exons with different boundaries (Table S3B). The corresponding protein coding potential resulted in 7 different complete isoforms (coding for proteins; Table S3D) from the 6 spliced and one unspliced mRNAs. The remaining left over mRNA variant (spliced) appears not to encode for a protein (non-coding). Similar structural features have also been documented (data not shown) at ASTD 1.1 [90]. According to AceView, this gene is expressed at high level in a wide range of tissues (Table S3B) revealing its heterogeneity of SCF expression, for example in human placental tissue, five SCF mRNA transcripts were detected [123] by RT-PCR, and that they appear to be under tissue-specific regulation whereas only one transcript size was detected in porcine endometrial total cellular RNA (tcRNA) [101].

In human, exon 1 (198 bp) is organized into 183 bp as 5′ UTR sequences and the last 15 bp including the initiation codon ‘ATG’ encode for the first 5 aa of the putative 25 aa signal peptide. Exons 2–7 encode portions of the extracellular domain of the SCF and exon 7 encodes the transmembrane region. While exon 8 encodes 35/36 aa of the cytoplasmic tail, the stop codon, and part or all of the very long ∼4.4 kb 3′ UTR (exon 9/10) of the SCF mRNA transcript [100]. As noted previously, SCF can exists as two alternative mRNA transcripts that have been identified for the presence (+) or absence (−) of the 84 nt sequences encoding the proteolytic cleavage site relative to the full-length SCF cDNA [5], [6], [25], [100]. Based on this SCF has been basically classified into variant-1 (+) and variant-2 (−) which are encoding for the protein 273 or 274 aa and 245 aa, respectively. The end points of the missing sequence correspond to the boundaries of exon 6 reported for the rat and human SCF genes [100]. This spliced feature is commonly seen in almost all vertebrate species (source: Ensembl). In murine, SCF cDNA (MGF94), the deletion in the second variant is smaller (48 bp) but shares the same 5′ boundary [5], this might be a due to different exon/intron structure for the mouse SCF (MGF) gene, or different alternative splicing within exon 6 that may have occurred during mRNA processing [97]. After analyzing the exon 5-intron (5)-exon 6 boundaries, it is certain that these transcripts are derived from the use of alternative 3′ splice donor/acceptor sites in the precursor mRNAs. The exon 6 region encoding for the 28 aa proteolytic site of oSCF is absolutely conserved (100% except for marmoset which has 92% identity) among the reported vertebrate SCF sequences (Figure 6(d); see also Figure S3(b)), suggesting a functional importance of this region of the molecule. However, avian species which has an additional 6 aa ‘SIGSNT’ (Figure S3(b) in a total of 34 aa shows 75% identity to its counterpart of 28 aa proteolytic site of other SCF (+) mRNA species. At this junction, fish has only 10–17% identity, revealing its long distance of evolutionary conservation for adaptation.

Scanning SCF gene through various genomes such as cow chr 5 (Btau_5.2, ENSBTAG00000017549); pig chr 5 (Sscrofa9, ENSSSCG00000000922); dog chr 15 (CanFam2.0, ENSCAFG00000006091); horse chr 28 (EquCab2, ENSECAG00000000152); human chr 12 (GRCh37, ENSG00000049130); chimpanzee chr 12 (CHIMP2.1, ENSPTRG00000005281); orangutan chr 12 (PPYG2, ENSPPYG00000004816); marmoset chr 9 (C_jacchus3.2.1, ENSCJAG00000019661); alpaca scaffold_157 (vicPac1, ENSVPAG00000006812); mouse chr 10 (m37, ENSMUSG00000019966); rat chr 7 (RGSC3.4, ENSRNOG00000005386); rabbit chr scaffold_18 (oryCun2, ENSOCUG00000017687); chicken chr1 (WASHUC2, ENSGALG00000011206); zebra finch chr 1A (Tae_Gut3.2.4, ENSTGUG00000008039); and zebra fish chr 25 (Zv8, ENSDARG00000070917 and ENSDARG00000058042), showed the overall expected size of SCF mRNA/cDNA for the (+) and (−) form ranging between 1.5 kb to 5.6 kb from cow, horse, dog, cat, pig to goat, human, mouse and rat. Of which the 3′ UTR sequence alone ranges between 490 bp to 4.4 kb. The longest ∼4.4 kb 3′ UTR mostly reported in brain, placenta, prostate, ovary and hematopoietic stem cells (source: GenBank, NCBI). Taking all the above into consideration, we have performed the 3′ RACE with high Tm primers, with or without DMSO in order to denature the suspected secondary structure [46] of the cDNAs and to enhance the target amplification. We also checked the presence of a wide range of SCF 3′ UTR amplicons up to 5 kb by performing a two-step PCR with a coupled annealing and extension time up to 10 min. We did not obtain the expected longer ∼4.4 kb as of different trials of 3′ RACE PCR amplification which inturn excluded the presence of such long 3′ UTR transcript variant (as in case of other mammals: AB002152.1, brain, goat; NM_000899.3, NM_003994.4, CR749222.1, Ref.Seq. annotated, human; XM_509255.2, Ref.Seq. annotated, chimpanzee; NM_013598.2, Ref.Seq. annotated, mouse; NM_021843.3, NM_021844.1, Ref.Seq. annotated, rat) in the skin of sheep. From the available database sequences and reports, we observe that the longest 3′ UTR is expressed in a tissue or cell type specific manner for the stable expression of SCF. Furthermore, the existence of such long 3′ UTR enhances the SCF mRNA structural stability and thereby regulates SCF expression (by miRNAs) which is required by the specific type of cell for its biological function [5], [124].

Overall, it was determined that the longer ovine s-SCF *isoform-1* (+) of 1519 nt which would encode for a larger secreted protein product of 274 aa (Figure 5(a)). This longer transcript has an insertion of 84 bp at nt. pos. 713–796 by an AS event corresponding to the 28 aa putative proteolytic cleavage site. Similarly, the novel, shorter ovine m-SCF *isoform-2a/2b* (−) of 835/725 nt (named in respect to the 5′ UTR differences; Figure S4(a_1_)) identified in this study would encode a smaller membrane-anchored protein product of 181 aa, which lacks the proteolytic site (Figure 5(b)). The mRNA/cDNA structural coverage of oSCF is shown in Figure 1A,B. The nucleotides and subsequent deduced amino acid sequences of the ovine SCF isoform (+/−, complete) has high % identity with other mammalian SCF species (Figure 6(a)). The longer and shorter oSCF cDNAs has 85–100% identity to the *kit ligand* ESTs which are deposited in mouse (DV046036.1, DV044494.1, DT909652.1) and human (DR005930.1, DR002356.1, BX474960.1, DC320486.1) especially in brain, prostate and hematopoietic stem cells but not in skin. In comparison to the previous submitted records, GenBank Acc. No. AAB49491.1 and the Swiss-Prot ID. P79368.2, our ovine s-SCF (+) form (GU386372; see Table S1) encoding for a total of 274 aa (Figure 5(a)), has an additional 7 aa i.e., ‘EREFQEV’ at its C-terminus (Figure S1B). Also, it differs from the Acc. No. CAA90620.1 with an additional 72 aa right after the proteolytic site, towards the C-terminus. Conversely, the alternatively spliced truncated transcript of ovine m-SCF (−) form (GU386373, GU386374 and GU386371; see Table S1) reported in this study has been recognized as novel, with a new additional 6 aa residues i.e., ‘KTYKHS’ as its C-terminus (Figure 6(c); see also Figure S1B and S3(c)), right after D^175^G. This ovine m-SCF (−) form completely lacks the proteolytic site, a transmmebrane region and the cytoplasmic tail (Figure 5(b)), in contrast to the 245 aa SCF (−) form that has been widely reported in other mammal species. Both soluble and transmembrane forms of SCF are active in promoting mast cell proliferation [5], [122]. However, the transmembrane form appears to be more potent in maintaining the viability of primordial germ cells *in vitro* [125]. Mice that produce the soluble SCF (s-SCF) but not transmembrane SCF (m-SCF) suffer from anemia, lack pigmentation and are sterile [126]. This suggests that transmembrane SCF plays a special role *in vivo* that is separate from that of soluble SCF. Hence, the presence of both soluble and transmembrane SCF is required for the normal biological function. The proteolytic processing can also occur in mouse SCF at a secondary site at or near the tetra-peptide ‘KAAK’ in exon 7 [10]. This secondary proteolytic cleavage site appears to be species-specific as in case of human there is an amino acid sequence divergence in this (‘KAKN’) region directing no protein processing [10]. The oSCF may also lack this secondary processing site as the amino acid sequence differs by ‘KASN’ from the mouse in that region (Figure S3(d)).

The ovine s-SCF (+) *isoform-1* specific 5′ RACE amplification (Figure 1A(c)) yielded a 364 bp amplicon with its isoform specific primer pair (Table S2) which is highly conserved among other mammals (Figure S4(a_2_)). Conversely, the common CDS region (+/−) primers (Table S2) yielded two different amplicon of sizes 325 bp and 215 bp (Figure 1B(d)) for the 5′ RACE RT-PCR which are subsequently differentiated by their 5′ UTR differences (Figure S4(a_1_)) and characterized as ovine m-SCF (−) *isoform-2a/2b*, respectively (in this study). All three 5′ RACE amplicons differ by their length for the (+) and (−) form as shown in Figure S4(a_1_). Owing to its high G+C content (65%; Figure S4(a_3_)), sheep SCF mRNAs has the potential to form compact, thermodynamically stable secondary structures (Figure 8(a,b)), due to the third hydrogen bond in G–C pairs compared to A–U pairs, and the ability of guanine residues to interact with uracil in folded RNA [127]. Henceforth, it favors the amplification of minor oSCF 5′ RACE cDNA products (in our case, *iso-2b* (−); Figure S4(a_1_)). The elevated G+C content is predicted to affect folding of the cDNA templates, compromising DNA polymerase processivity [111]. G+C sequence bias is a well known problem in cDNA profiling studies [128]. This is not only because of the fall out of *Taq DNA polymerase* during PCR, also at certain level of reverse transcription by reverse transcriptase since our sequenced individual clones of all three 5′ RACE products (+/−) has the complete 5′ adapter forward primer sequences complementary to the 5′ end capping (C-tail). The GC-rich non-coding 5′ segment of SCF forms a dense secondary structure (Figure 8(a,b)) that may have the consequences for oSCF protein expression. For example, translation may require specific mRNA unwinding activity, creating another mode of possible post-transcriptional regulation [129]. Furthermore, mRNA hairpin structures are known to obstruct ribosome elongation [130] and G+C content is inversely correlated with translation efficiency [131].

Apart from the classical 273 or 274 aa SCF starting with ‘MKK’ as its N-terminus sequences, there are a number alternatively spliced protein/peptide sequences do exists for SCF, resulting in a unique or skipped N-terminus sequences such as, N-terminus starting with ‘MPSCLAAQ’ (protein: CAH18078.1, peptide: ENSPTRP00000050003, ENSCJAP00000036530: 238 aa) in human, chimpanzee and marmoset, respectively; ‘LFKTL’ (peptide: ENSCJAP00000036543: 273 aa) in marmoset; ‘LLKTL’ (peptide: ENSP00000349630, ENSPTRP00000045663: 273 aa); ‘TWII’ (peptide: ENSECAP00000000163, ENSCAFP00000009149: 269 aa) in horse, dog, respectively; ‘LLFN’ (protein: AAM16280.1: 258aa) in dog; ‘LQPS’ in cow (peptide: ENSBTAP00000023349: 212 aa); ‘ICRNR’ (peptide: ENSSSCP00000000985: 177 aa) and ‘TWIIT’ (peptide: ENSSSCP00000000986: 38 aa) in pig; ‘KKKE’ (peptide: ENSRNOT00000008471: 272 aa) in rat and ‘IITC’ (protein: AAB49491.1: 260 aa) for sheep. In mouse, SCF has 6 to 9 different GenBank/Ensembl records with the minimum of peptide containing 123 aa residues up to protein of 273 aa residues including an unique N-terminus sequence of ‘NRTE’ (peptide: ENSMUSP00000100919, ENSMUSP00000100918). This kind of alternatively spliced N-terminus do exists even in birds and fishes i.e., ‘FFTKQ’ (peptide: ENSTGUP00000008290: 287 aa) in zebra finch; ‘GNPV’ (protein: ABI98396.1: 264 aa and ABI98398.1: 164 aa) in chicken; ‘MTGF’ (protein: XP_002666882.1: 292 aa) and ‘IWIC’, ‘MFHM’ (peptide: ENSDARP00000101828, XP_682759.2: 267 aa) in zebra fish. The above collective details are obtained from Ensembl and GenBank, NCBI.

Consistent with the already reported and submitted SCF sequences, oSCF gene consists of 9/10 exons separated by 8/9 introns (Figure 3(e,f)). Exon sizes correlate well with those reported for the human, mouse and dog (source: GenBank, Ensembl). From the gDNA spliceosomal intron-5 amplification, the premature termination could be explained by the use of an alternative isoform/cryptic 5′ donor site at nt pos. 218 (GT, Figure 6(b) and S4(b); right after 57 nt of the p(A)_11_) and a constitutive 3′ acceptor (AG) at nt pos. 728 (just before the start of exon 6) or the one at nt pos. 350 recognised by the transcription machinary (Spliceosome) and/or the lack (?) of any consensus 3′ splice site sequence downstream of exon 6 to exon 9/10 prevents the removal of the 161 nt intronic sequences which is present in the shorter cDNA (Figure 2(a,b) and S4(c)). The retaining of 161 bp noncoding DNA (intron-5) sequences in the truncated shorter m-SCF (−) cDNA might have arisen from failure of the transcription machinary to correctly remove the intronic sequence from the skin oSCF mRNA transcript. Though the chromosomal number was determined in sheep (chr 3) [105], it was observed that the sheep SCF locus is yet to be mapped (see Figure 4), depicting its unfinished status of the Sheep Genome Project at this juncture (current version Oarv2.0, March 2011 - till date, http://www.livestockgenomics.csiro.au/sheep/oar2.0.php). The mechanism illustrated in Figure 3(c,d) (for splicing notation) explains how the truncated oSCF mRNA could have been generated in the normal skin and adds to the list of variants of the SCF gene that undergo alternative splicing (AS).

Previous studies have shown that skin expression of SCF stimulates melanocyte migration, proliferation, differentiation, and survival and is required for ongoing maintenance and survival of normal melanocyte numbers in adults [132]. SCF (KL) upstream region is associated with significant differences in human skin color, one of the most obvious superficial differences between human populations [133]. Although no amino acid differences are known in the SCF (KL) protein of different human groups, SCF is expressed at significantly higher levels in skin keratinocytes from Africans than Europeans [134]. The interruption of SCF–KIT signalling using anti-KIT antibody abolished tyrosinase and MITF expression, resulting in the depigmentation of hair follicles in a reversible manner [16].

The preliminary analysis of oSCF gene expression in skin, showed similar mRNA (cDNA) expression profile between (+) and (−) form among white and coloured animals (data not shown). Our result was in agreement with porcine SCF (KL) gene expression for exon 6 [121]. However, this would require verification via more sensitive qRT-PCR methods on reasonable number of breeding populations i.e., F_2_ generations. Conversely, Northern blot analysis (Figure 7) revealed considerable difference between oSCF (+) and (−) form providing a hypothetical clue on transcription regulation via an intron-5 AS event. Different biological activity have been reported between the membrane anchored (−) and the soluble forms (+) of SCF [9], [11]. In 1999, Dr. James M. Grichnik, wrote in his reply to [135] “*While both forms of SCF activate its receptor, KIT, the duration of activation and potential for receptor degradation is different for each form. Keratinocytic bound SCF may lock on to the melanocyte’s KIT receptor resulting in persistent KIT activation (without KIT receptor internalization and degradation), while soluble SCF may transiently activate the KIT receptor followed by internalization and degradation”.* This implies that the membrane-bound steel factor induces more persistent tyrosine kinase activation and longer life span of c-KIT gene-encoded protein than its soluble form. More sustained signaling was mediated by membrane associated SCF in a myeloid cell line where as the soluble SCF down regulates cell surface expression of c-KIT and promotes receptor proteolysis [136]. The differential expression of SCF-specific mRNA splice variants, SCF-1 and SCF-2 in immature and mature human mast cells may play a role in autocrine stimulation, maintenance of survival and the differentiation of tissue mast cells [137]. An increased level of soluble SCF expression in the skin has been implicated in the pathogenesis of mastocytosis that could theoretically be due to the abnormality at any level of metabolism occurring after the mRNA transcription and splicing rather than the result of changes in the sequence or regulation of the gene itself [19]. Hence, further investigation regarding sheep skin SCF gene expression is required at cellular level rather than at tissue basal level. The possible functional role of these two oSCF isoforms in skin remains poorly understood. According to AceView [89] gene expressinon analyses, SCF is defined by 198 GenBank accessions from 192 cDNA clones, some from brain (seen 14 times), trachea (13), placenta (9), thalamus (7), whole brain (7), lung (6), amygdala (5) and 61 other tissues excluding skin. Molecular biological analyses of murine follicular skin indicated a significant increase of membrane-bound SCF expression [16], after anagen induction in concert with the escalation of cutaneous tyrosinase activity and corresponding pigmentation.

Eukaryotic splicing produces a variety of functional and nonproductive mRNAs during normal gene expression [138]. While alternative splicing greatly enhances recurrent errors that include exon skipping, intron retention, and activation of cryptic splice sites [138]. The resulting aberrant RNAs may outnumber correctly spliced mRNAs among initial spliceosomal products [139]. This could be one of the reason for the oSCF (−) form to be present predominant over (+) form during the reverse transcription reaction (RT) and its subsequent PCR amplification. For protein-coding genes with multiple exons, the majority of aberrant RNAs contain a premature truncation codon (PTC; in our case, the shorter ovine m-SCF (−) form) which are frequently produced in mammals are known to be degraded through the nonsense-mediated decay (NMD) pathway [140]. However, the abundance of full length oSCF (−) mRNA transcripts in the skin of sheep argues against such degradation.

Control of gene expression is achieved at various levels. The *cis*-regulatory elements, uORFs (in + and - form) and TOP (in + form) detected on the 5′ UTR of oSCF just upstream to the AUG initiation codon (Figure S4(a_1_)) are known to be involved in the translation down regulation. The uORFs can induce formation of a translation-competent ribosome that may translate and (i) terminate and re-initiate, (ii) terminate and leave the mRNA, resulting in down-regulation of translation of the main open reading frame, or (iii) synthesize an N-terminally extended protein [108]. The 5′ TOP tract consisting of 5–15 pyrimidines that is required for coordinate translational repression during growth arrest, differentiation, development and certain drug treatments [141]. Deletion of the pyrimidine tract or exchanging purines for pyrimidines results in unregulated translation [109], [141]. In our case, we observed the deletion of TOP sites in the two shorter 5′ UTRs of oSCF-*2a/2b* (−) form (Figure S4(a_1_)). Regarding the 3′ UTR *cis*-regulatory sequences such as AREs (PAS) [110], BRD-Box [111] and MBE [112] mediates negative post-transcriptional regulation by affecting mRNA transcript stability and translational efficiency [110], [140]. In our case, the 3′ *cis*-regulatory signals, BRD-Box and MBE, located upstream and downstream PAS (Figure S4(d,c)) may regulate tissue-specific alternative polyadenylation which has been detected in approximately 54% of human genes [142]. The exact role of the conserved miRNA target sites (Figure 9(a,b)) in SCF is currently unknown, although this conservation in other farm animals (71–100%) suggests functional importance (evolutionary pigmentation adaptation). On the other hand, various miRNA target sites in the longer 3′ UTR (data not shown) might signify that the mRNA is regulated specifically in different tissues or at different times during development. The potential role of miRNAs in SCF gene regulation is currently unidentified in particular for hair follicle melanogenesis.

SCF is a member of the helical cytokine structural super-family characterized by a double crossover four-helix bundle topology [143]. The N-terminal 141 residues of SCF have been identified as a functional core, SCF^1−141^, which includes the dimer interface and portions that bind and activate its receptor, c-*kit* [112]. The homology-based structural modeling results showed that the protomer structure of oSCF contained 4 α-helices and 2 β-sheets that were folded to form the non-covalent homodimer composed of two slightly wedged protomers [114]. The two disulfide bridges between Cys^29^/Cys114 and Cys^68^/Cys^164^ (Figure S5) plays a role in maintaining the functional integrity of SCF [143] and are highly conserved in mammals except for fishes where it is replaced with Ile^27^/His^107^ (Figure S3(d)). The available PDB crystallographic models for SCF proteins such as 1EXZ, 1SCF and 2E9W:chain C, D [112]–[114] share the same canonical fold. The superimposition of our modelled structure(s) to the individual templates revealed identical structural features as described in [112]. The folding differs in some regions from the above mentioned models with an additional 3 or 4-turn helices as depicted in Figure S5. The previously determined crystal structure 2E9W, demonstrates the interaction between SCF and its receptor, c-KIT [114]. In which, each protomer of SCF binds exclusively to a single KIT molecule and that receptor dimerization is driven by SCF dimers that facilitate additional receptor-receptor interactions. Dimerization of KIT is driven by bivalent SCF binding whose sole function is to bind SCF and to bring together two KIT molecules [114]. The three potential binding region of SCF i.e., site I, II, III for its receptor, c-KIT has been well explained in [114] and the same are shown in Figure S5 (see also Figure S3(d)). There are notable differences found in the interacting residues of KIT and SCF [114]. Mutational analysis of SCF has shown that replacement of Asn^35^ with alanine or glutamic-acid residue, reduces the binding affinity of SCF towards KIT by approximately 10-fold and Asn^35^ (in human, chimpanzee and marmoset) or Asp^35^ (in other species) is required for the biological activity [144]. ClustalW comparison (Figure S3(d)) of the receptor-binding interface in SCF from different species shows the high conservation for Asn^35,36^ in human, chimpanzee, marmoset or Asp^35,36^ in sheep, goat, cow, pig, dog, panda, cat, horse, chicken, zebra finch, zebra fish and gold fish and Asp^35^, Asn^36^ in mouse, rat and rabbit (Figure S5). Similarly, Asp^79^ of SCF in human, chimpanzee is substituted by a Leu^79^ in mouse or Val^79^ in sheep, goat, cow, pig, dog, panda, cat, horse, rabbit, rat, zebra fish and gold fish or Ser^79^ in marmoset, chicken and zebra finch. Besides, Lys^106^ in sheep, goat, cow, pig, dog, panda, cat, horse, mouse, rat and rabbit is substituted by Asn^106^ or Arg^106^ in human, chimpanzee, marmoset and chicken, zebra finch respectively. In addition, Glu^113^ of SCF in sheep, goat, cow, pig, dog, panda, cat, horse, rabbit, human, chimpanzee, marmoset, is substituted for by Leu^113^ and Ala^113^ residues in mouse and rat, chicken, zebra finch, respectively. Similarly, Phe^127^ (loss of a hydrogen bond) in human, chimpanzee, marmoset is substituted with Ser^127^ in sheep, goat, cow, pig, cat and rabbit which is quite common in protein fucntional centres, most likely able to form a hydrogen bond. All these substitutions (Figure S3(d)) involved in salt bridges, hydrogen and van-der-Waals bonding may account for the reduced affinity of SCF towards its receptor, c-KIT [145].

SCF (KITLG) was found not only in the mammal species such as sheep, goat, cow, pig, cat, dog, panda, horse, human, chimpanzee, marmoset, mouse, rat, and rabbit but also in avian such as chicken, zebra finch and fishes, such as zebra fish, gold fish, indicating that it had the co-emergence with huge divergence across species (Figure S6(a-e)). The enormous evolutionary distance on the phylogentic tree (branch length) indicate the low sequence identity of the fish (Figure 6(a); see also Figure S3(d)) species to the other mammal species ranging between <20–55% for the SCF (+) and (−) protein sequences which implies SCF evolutionary changes may make it as monophyletic group(s) for more pigmentation adaptation in a wide range of habitats. One such example is that, the *cis*-regulatory (UTRs) changes in SCF (KL) expression contribute to pigmentation differences in both sticklebacks and humans suggesting its contribution to natural variation in vertebrate pigmentation and those similar genetic mechanisms may underlie rapid evolutionary change in sticklebacks and humans to rapidly evolve changes in pigmentation patterns [146]. The little skate in the tree topologies (Figure S6(a-e)) especially those nodes showing <60% bootstrap values viz. horse to dog; mouse, rat to rabbit; and rabbit to primates are most likely reflects the use of incomplete SCF sequences from the gene/genome databank (partial sequences, unfinished genomes) or due to the use of unwanted gaps in the alignment or could be the huge sequence divergence at certain level in the block analyzed in the present study.

### Conclusion

The study that we describe here represents the first attempt to experimentally address the SCF mRNA/cDNA structural coverage in the skin of merino sheep. The analysis of coat color gene(s) structure unique to sheep will extend our understanding of the functional role and regulation of pigmentation genes beyond what was known in mice, humans and other mammals. Here, we have presented evidence for two splice variants of ovine SCF, differing in the cassette exon (CE 6–9/10; skipping of exons 6–9/10) by a premature termination in the non-coding intron 5, which resulted in the presence or absence of a proteolytic site and there by the following transmembrane region and cytoplasmic domain. To our knowledge, this information is previously unreported. Further research is required to determine whether this premature terminated isoform has biological relevance, and whether it leads to the active variant proteins with effects on melanocytic, reproductive or haematological development. The functional role of these two transcripts in ovine skin-specific expression remains unknown. It is important to elucidate which SCF transcript(s), either soluble-SCF (+) or membrane-SCF (−), predominate in the skin which will provide a new insight into an elaborate mechanism involving m-SCF/c-KIT and its counteracting s-SCF/c-KIT signaling that will add to the understanding of the regulation of SCF on hair follicle melanogenesis. In addition, characterization of the SCF promoter(s) is also critical to the design of experiments intended on analysis of the role of various SCF isoforms *in vivo* using gene targeting techniques. Also, in connection to [33], it would be interesting to determine whether any of the individuals (white, black, and brown) in their families (F_2_ generation) have alterations in the SCF gene expression at allele level (QTL/SNPs) or it may have the other alternative splice variant(s)? or lacking any particular reported SCF variants or duplication [147] and/or SCF DNA rearrangement [148]. Future studies exploring other candidate genes are underway especially those involved in the pigmentation regulatory network namely c-KIT and MITF. Altogether, these genes are likely to provide great insight into our understanding of molecular mechanism of the white trait in merino sheep. In this context, further developing ovine chip(s) with key pigmentation associated genetic information such as c-KIT, SCF, MITF, MC1R, ASIP and FGF etc., will open up promising perspectives on using those molecular information in the management of breeding schemes of sheep populations i.e., aiming at Gene Assisted Selection (GAS).

## Supporting Information

Figure S1 **SCF Multiple Sequence Alignments (MSA). (A)** Comparison of the primary RT-PCR product of 621 bp CDS covering the putative primary proteolytic site of white, black and brown animal (representative data from one of three animal is shown). The start (ATG) codon is labeled in bold blue letters and the +84 bp proteolytic site is indicated in bold black italic letters. The virtual translation of 606 bp CDS corresponding to the 202 aa (in bold black letters) is given below to the ‘white’ nucleotide sequences; **(B).** Comparison of complete coding sequence (CDS) and its corresponding deduced amino acid sequence of the newly isolated *Ovis aries* SCF *isoform-1* (+) and *isoform-2* (−) with the partial GenBank records of oSCF (+) sequences. The newly identified oSCF cDNAs from the skin of white merino sheep (GU386372 (+); GU386373 (−), see. Table S1) are marked in bold black letters. While the other two oSCF partial CDS sequences (U89874.1; Z50743.1) retrieved from GenBank, NCBI. Dotted black arrows indicate the corresponding common forward primer ‘scffwd1’ and (+) form specific reverse primer ‘scfrev1’ used to amplify the initial 621 bp (see also Figure 1A(a)). The highlighted opened black box indicates the flanking partial 5′ UTR sequence (15 bp) of the forward primer sequence (see. Table S2 and Figure 1A). The start (ATG) and stop codons (TAA) are labeled in bold blue and bold red letters respectively. The virtual translation of oSCF (+) and (−) forms are given below to the respective triplet codons and highlighted in bold black letters. The +84 bp putative primary proteolytic site and its virtual translation (+28 aa) are indicated in bold black italic letters. Similarly, the substitution of aspartic acid (D) with glutamic acid (G) i.e., D(+)^175^G(−) is indicated in bold light orange to bold light green letters respectively (see the chromatogram of cDNA on the left side). The new truncated protein isoform of oSCF (−) form having a short stretch of 6 aa sequences as its C-terminus (in bold black letters) is highlighted by opened green box. Two clone differences are highlighted in bold red and bold light blue letters (see the respective cDNA chromatograms given on left side).
(DOC)

Figure S2 **AceView of human SCF (KITLG) gene encoded on minus strand of chromosome 12 (huchr 12).** Alternative mRNAs shown are aligned from 5′ to 3′ on a virtual genome where introns (triangle lines in pink) have been shrunk to a minimal length. Exon size is proportional to length (shaded and opened square/rectangle pink boxes; see key to symbols), intron height reflects the number of cDNA clones supporting each intron. The mRNAs/cDNAs appear to differ by truncation of the 5′ and 3′ end and by the presence (+) or absence (−) of 84 bp insertion for the proteolytic site (shaded in light green between exon 5/exon 6 on AceView variant b, a). In the above diagram, capped 5′ ends and aggregated 5′ clones are indicated by shaded and opened black tower pointers respectively. Similarly, validated 3′ ends with varying number of accessions (clones) are indicated by opened and shaded blue, black tower pointers. Alternatively spliced (gt-ag) introns are shaded with four different colours (light green, lavender, light blue and light yellow).
(DOC)

Figure S3 **Amino acid (aa) sequence conservation of sheep SCF with other homologous vertebrate SCF.** **(a)** Conservation of the first 25 aa signal peptide of sheep SCF is shown along with other species; **(b)**, **(c)** Comparison of s-SCF and m-SCF; **(b)** Highlights the conservation of the 28 aa proteolytic site right after D^175^ (in blue bold letters) is indicated in black bold letters where in the additional 6 aa avian sequences are indicated in red bold letters along with other aa substitutions indicated in black bold letters; **(c)** The novel C-terminus end of sheep m-SCF (in this study) right after G^175^ (in red bold letters) is indicated in green bold letters (final 6 aa sequences) and its alignment with other predicted m-SCF C-terminus sequences are shown. In all the cases, sheep SCF aa sequences are compared mainly with the human SCF aa sequences, hence both are highlighted in black bold letters; **(d)** The main alignment block showing topological features of sheep s-SCF such as four α helices and two β sheets are shown. In addition, ClustalW2 comparisons of the three potential receptor (c-*kit*) interactive sites (Site I, II and III) in SCF from different species [114] are shown. Sheep s-SCF orthologous evolutionary aa substitutions are highlighted in black bold letters. The four cysteine residues involved in disulfide bridges are indicated in pink bold letters and the orthologous aa substitutions in avian species are highlighted in red bold letters. An additional aa residue at Glu(E)^155^ in sheep s-SCF which differentiate it from primates and rodents is highlighted in blue bold letters, which is conserved in farm animals suggesting a functional importance of this residue. Besides, the 28 aa proteolytic site, the putative alternative proteolytic site, a tetra peptide [10] is indicated in black bold letters.
(DOC)

Figure S4 **Nucleotide sequence comparison of 5′ and 3′ untranslated regions (UTRs) of sheep SCF** ***isoform-1*** **(+) and** ***isoform-2a/2b*** **(**−**), the predicted UTR regulatory motifs and the possible splice donor/acceptor sites on intron-5 are shown.** **(a_1_)** Sequence alignment shows sheep SCF 5′ UTR length differences between *isoform-1* (+) and *isoform-2a/2b* (−). The additional sequences of 5′ UTRs are indicated in green, light orange and blue opened boxes for *isoform-1* (+), *isoform-2a* (+) and *isoform-2b* (−), respectively. The *cis*-regulatory elements located in the the 5′ UTR such as TOP and uORFs are labeled and indicated in red opened boxes. The trinucleotide elements such as ‘CGC’ and ‘TGC’ are highlighted in bold black letters and by underline respectively. The hexamer direct repeats (DRs) are labeled and indicated by opened boxes. Clone differences are labeled in bold red to bold black letters; **(a_2_)** Alignment shows SCF 5′ UTR nucleotide sequence conservation of hexamer DRs (in opened boxes) with other mammals; **(a_3_)** Histogram shows the GC% of three different 5′ UTR of sheep SCF; **(b)** The complete sequence of sheep SCF intron-5 (729 bp) shows the constitutive splice donor (GT, in bold blue upper case letters) site at the start and the constitutive splice acceptor (AG, in bold red upper case letters) site at the end. Other alternative isoform/cryptic splice donor (gt), acceptor (ag) sites are labeled in blue, red lower case letters respectively. The dinucleotide repeats, polyA stretch (p(A)) and predicted splice branch sites (BS, in green lower case letters) are labeled and highlighted in opened boxes; **(c)** Nucleotide sequence alignment shows 100% similarity of 3′ UTR of *isoform-2* (−) with 161 bp retained intron-5 of sheep SCF. The p(A) stretch and the conservation of dinucleotide repeats flanked by two tandem repeats (TRs) on either side of 3′ UTR are marked in opened boxes along with its counterpart sequences on intron-5 in other animals; **(d)** Sequence alignment shows two different 3′ UTRs of sheep SCF *isoform-1* (+) and *isoform-2* (−). The +84 bp proteolytic site is indicated in bold black italic letters. The common identical CDS just upstream to the proteolytic site are indicated in opened box. The 3′ UTR regulatory motifs such as BRD, MBE and hepatamer DRs are labeled and highlighted with opened boxes. In the above figure, the AREs located in the 3′ UTR near by the canonical PAS are indicated by an underline and the single base variants of its type is highlighted in blue letters. Similarly, the start (ATG) and stop codons (TAA) are highlighted in bold blue and bold red letters respectively.
(DOC)

Figure S5 **Three-dimensional structure of oSCF monomer generated by homology-based modelling represented by a ribbon diagram.** Here the superimposition of oSCF monomer to the PDB template 1EXZ:chainB (set to transparency) is shown. The 4 α-helix, two antiparallel β-sheets, two additional one-turn helix are labelled in blue (αA, αB, αC and αD), red (β1, β2) and black (αB’, αD’) letters respectively. An exceptional hairpin loop between αB and αC is shown in red dotted line. The observed additional 3–4 turn helices are highlighted in green as G_1_ to G_8_ with the corresponding aa residues labeled respectively. The three potential interactive sites of SCF for its receptor c-*kit* are shown in bold letters as Site I, Site II and Site III [95]. In comparison to human SCF, the highlighted aa residue in red at Site I, II and III represents the orthologous substitution of aa residues in accordance with sheep, goat, cow, pig, dog, horse, cat and panda SCF to huSCF protein. The two disulfide bridges Cys^29^/Cys^114^ and Cys^68^/Cys^164^ are highlighted in pink.
(DOC)

Figure S6 **Phylogenetic analysis of the two SCF isoforms based on alignment of their complete nucleotide sequences (CDS), deduced amino acid sequences, and predicted DNA sequences representing exon(5)-intron-5-exon(6) splice junction of SCF gene.** Numbers on the respective nodes denote percentages in the order of Neighbour-Joining (NJ) using p-distance/Maximum likelihood (ML)/Bayesian (BI) posterior probabilities. The values in the tree nodes represent bootstrap values of 1000 trials, indicating the credibility of each branch. Branch lengths are proportional to the number of amino acid or nucleotide changes on the branch. **(a)** Phylogenetic tree inferred from 17 soluble SCF (+) protein sequences; **(b)** Phylogenetic tree inferred from 18 soluble SCF (+) nucleotide sequences; **(c)** Phylogenetic tree inferred from 13 membrane-bound SCF (−) predicted protein sequences; **(d)** Phylogenetic tree inferred from 14 membrane-bound SCF (−) predicted nucleotide sequences; **(e)** Phylogenetic tree inferred from 14 predicted DNA sequences representing exon(5)-intron-5-6-exon(6) splice junction (+/−) of SCF gene.
(DOC)

Table S1 **GenBank Accession Nos. and description of ovine SCF cDNAs submitted to NCBI.**
(DOC)

Table S2 **Details of Oligonucleotide primers used and the corresponding experiment/target amplification of ovine SCF isoforms.**
(DOC)

Table S3 (A) Molecular annotation of mRNAs, the pre-messenger or transcription unit, the 5 kb upstream and the UTRs for SCF (KITLG; source: Aceview*). (B) Comprehensive details of SCF mRNAs structure and different tissue expression (source: AceView). (C) Details of the validated polyA sites of SCF (source: AceView). (D) Details of the alternatively spliced, predicted SCF protein properties (source: AceView).
(DOC)
