# Supplementary figures and images for: Skipping of Exons by Premature Termination of Transcription and Alternative Splicing within Intron-5 of the Sheep SCF Gene: A Novel Splice Variant
Source: PLoS One. 2012 Jun 15;7(6):e38657. doi: 10.1371/journal.pone.0038657 (PMC3376141; doi:10.1371/journal.pone.0038657)

**Figure S1A**


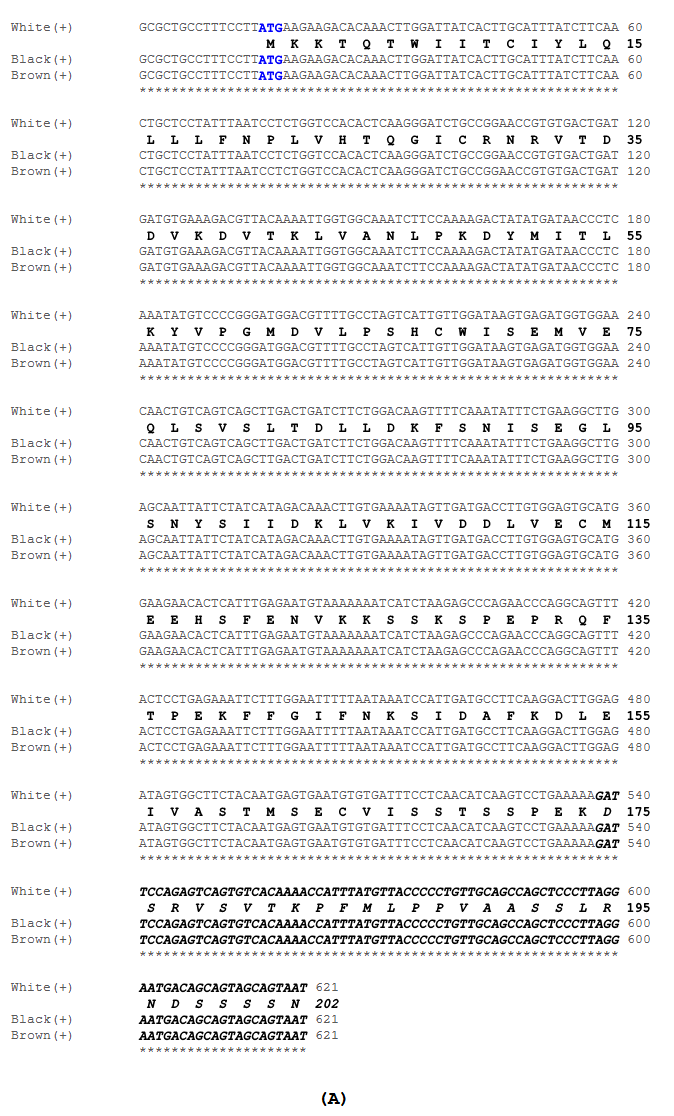


**Figure S1B**


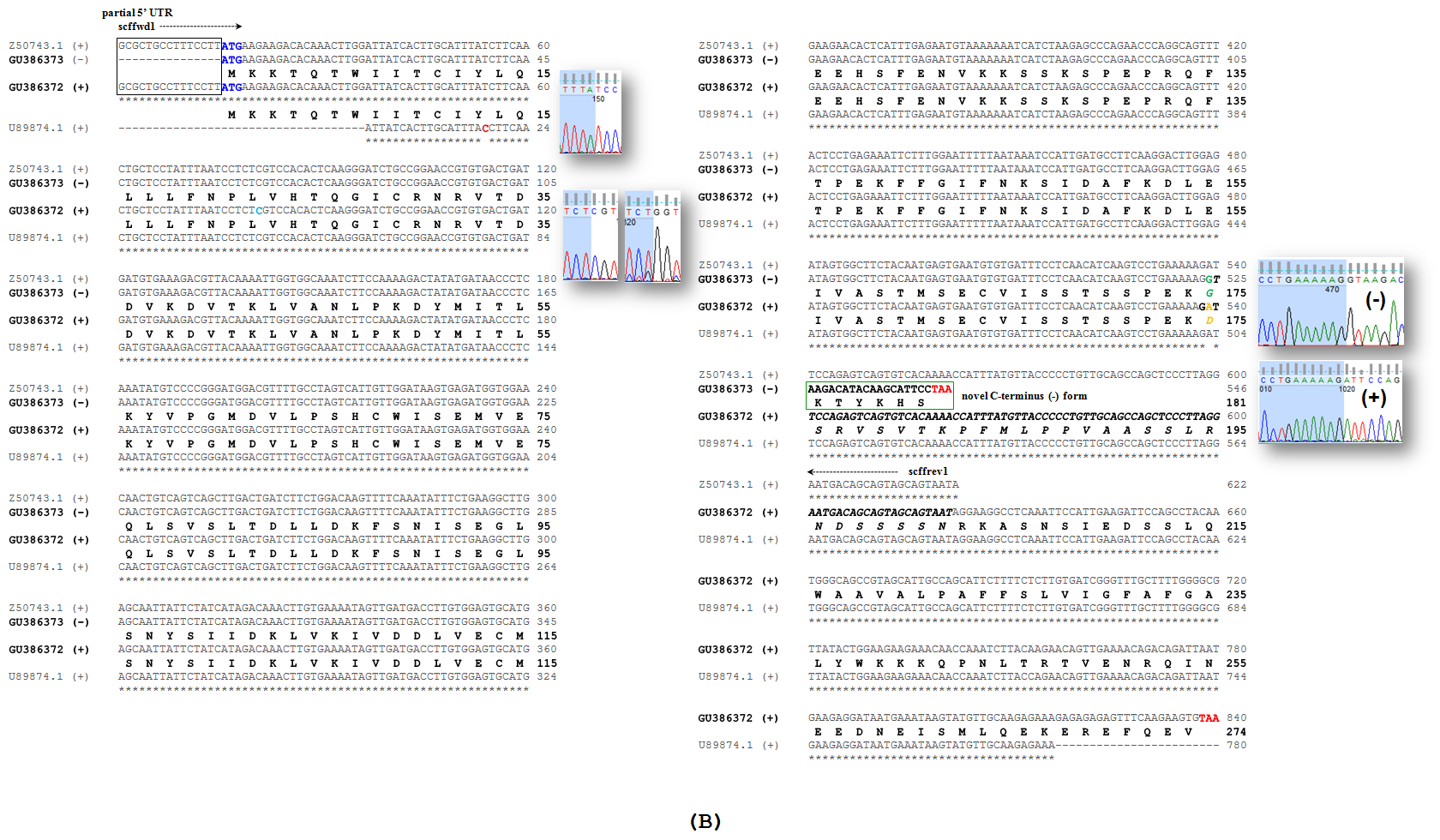

Supplement: Figure S1 — SCF Multiple Sequence Alignments (MSA). (A) Comparison of the primary RT-PCR product of 621 bp CDS covering the putative primary proteolytic site of white, black and brown animal (representative data from one of three animal is shown). The start (ATG) codon is labeled in bold blue letters and the +84 bp proteolytic site is indicated in bold black italic letters. The virtual translation of 606 bp CDS corresponding to the 202 aa (in bold black letters) is given below to the ‘white’ nucleotide sequences; (B). Comparison of complete coding sequence (CDS) and its corresponding deduced amino acid sequence of the newly isolated Ovis aries SCF isoform-1 (+) and isoform-2 (−) with the partial GenBank records of oSCF (+) sequences. The newly identified oSCF cDNAs from the skin of white merino sheep (GU386372 (+); GU386373 (−), see. Table S1) are marked in bold black letters. While the other two oSCF partial CDS sequences (U89874.1; Z50743.1) retrieved from GenBank, NCBI. Dotted black arrows indicate the corresponding common forward primer ‘scffwd1’ and (+) form specific reverse primer ‘scfrev1’ used to amplify the initial 621 bp (see also Figure 1A(a)). The highlighted opened black box indicates the flanking partial 5′ UTR sequence (15 bp) of the forward primer sequence (see. Table S2 and Figure 1A). The start (ATG) and stop codons (TAA) are labeled in bold blue and bold red letters respectively. The virtual translation of oSCF (+) and (−) forms are given below to the respective triplet codons and highlighted in bold black letters. The +84 bp putative primary proteolytic site and its virtual translation (+28 aa) are indicated in bold black italic letters. Similarly, the substitution of aspartic acid (D) with glutamic acid (G) i.e., D(+)175G(−) is indicated in bold light orange to bold light green letters respectively (see the chromatogram of cDNA on the left side). The new truncated protein isoform of oSCF (−) form having a short stretch of 6 aa sequences as its C-terminus (i [file pone.0038657.s001.doc]

**Figure S2**


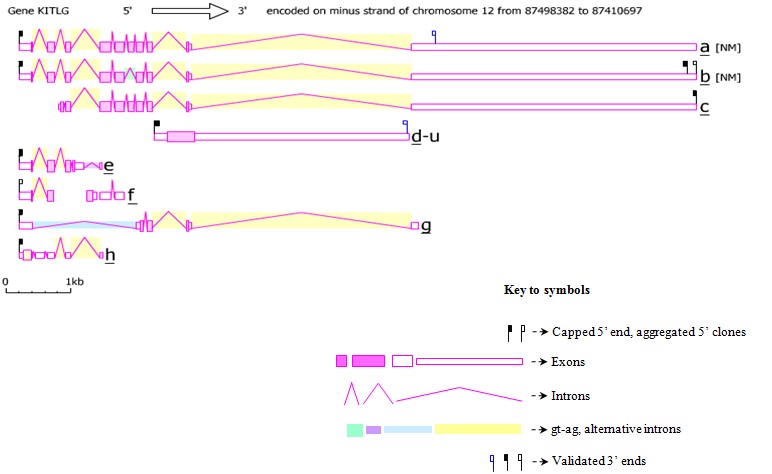

Supplement: Figure S2 — AceView of human SCF (KITLG) gene encoded on minus strand of chromosome 12 (huchr 12). Alternative mRNAs shown are aligned from 5′ to 3′ on a virtual genome where introns (triangle lines in pink) have been shrunk to a minimal length. Exon size is proportional to length (shaded and opened square/rectangle pink boxes; see key to symbols), intron height reflects the number of cDNA clones supporting each intron. The mRNAs/cDNAs appear to differ by truncation of the 5′ and 3′ end and by the presence (+) or absence (−) of 84 bp insertion for the proteolytic site (shaded in light green between exon 5/exon 6 on AceView variant b, a). In the above diagram, capped 5′ ends and aggregated 5′ clones are indicated by shaded and opened black tower pointers respectively. Similarly, validated 3′ ends with varying number of accessions (clones) are indicated by opened and shaded blue, black tower pointers. Alternatively spliced (gt-ag) introns are shaded with four different colours (light green, lavender, light blue and light yellow). (DOC) [file pone.0038657.s002.doc]

**Figure S3**


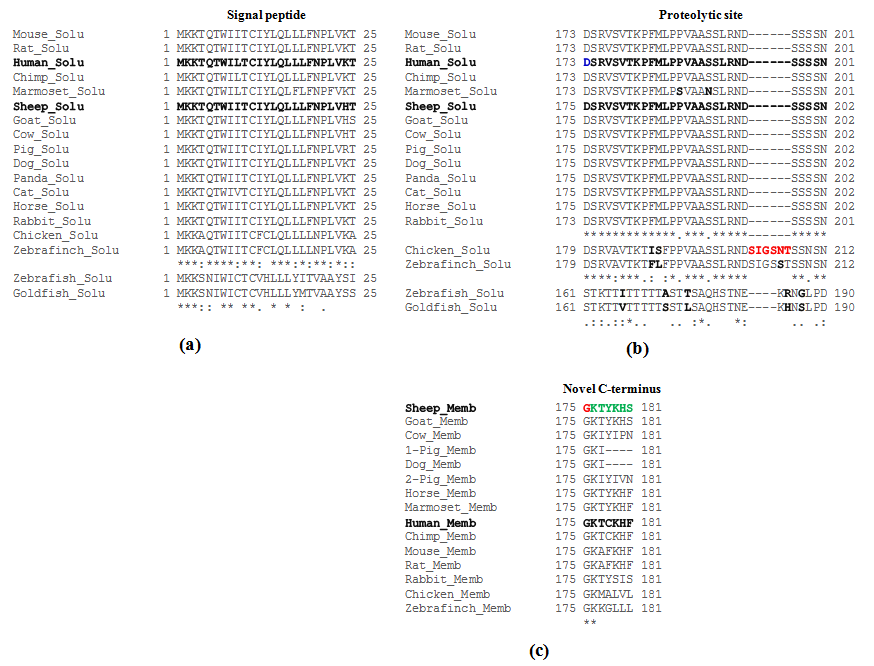


**Figure S3**


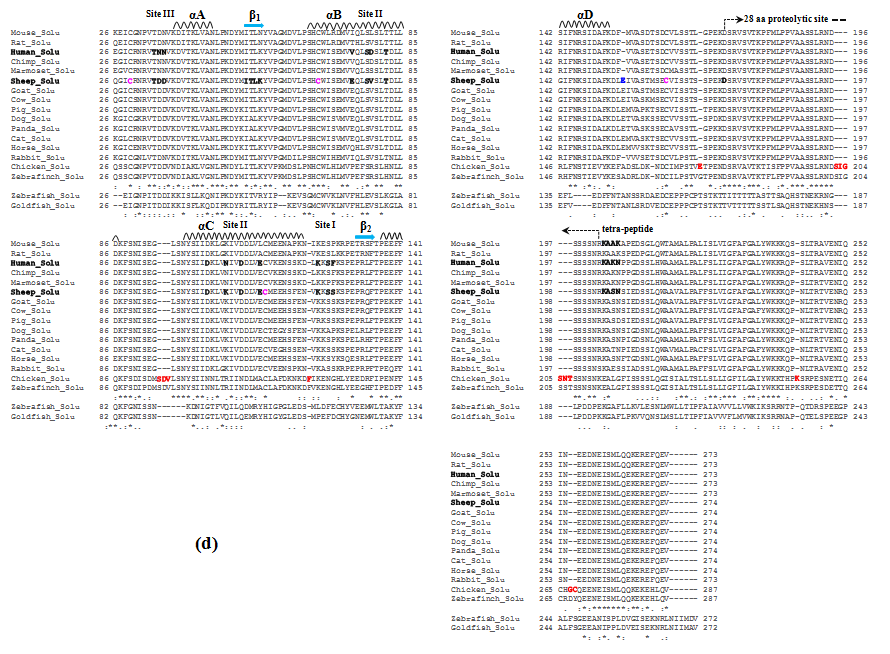

Supplement: Figure S3 — Amino acid (aa) sequence conservation of sheep SCF with other homologous vertebrate SCF. (a) Conservation of the first 25 aa signal peptide of sheep SCF is shown along with other species; (b), (c) Comparison of s-SCF and m-SCF; (b) Highlights the conservation of the 28 aa proteolytic site right after D175 (in blue bold letters) is indicated in black bold letters where in the additional 6 aa avian sequences are indicated in red bold letters along with other aa substitutions indicated in black bold letters; (c) The novel C-terminus end of sheep m-SCF (in this study) right after G175 (in red bold letters) is indicated in green bold letters (final 6 aa sequences) and its alignment with other predicted m-SCF C-terminus sequences are shown. In all the cases, sheep SCF aa sequences are compared mainly with the human SCF aa sequences, hence both are highlighted in black bold letters; (d) The main alignment block showing topological features of sheep s-SCF such as four α helices and two β sheets are shown. In addition, ClustalW2 comparisons of the three potential receptor (c-kit) interactive sites (Site I, II and III) in SCF from different species [114] are shown. Sheep s-SCF orthologous evolutionary aa substitutions are highlighted in black bold letters. The four cysteine residues involved in disulfide bridges are indicated in pink bold letters and the orthologous aa substitutions in avian species are highlighted in red bold letters. An additional aa residue at Glu(E)155 in sheep s-SCF which differentiate it from primates and rodents is highlighted in blue bold letters, which is conserved in farm animals suggesting a functional importance of this residue. Besides, the 28 aa proteolytic site, the putative alternative proteolytic site, a tetra peptide [10] is indicated in black bold letters. (DOC) [file pone.0038657.s003.doc]

**Figure S4**


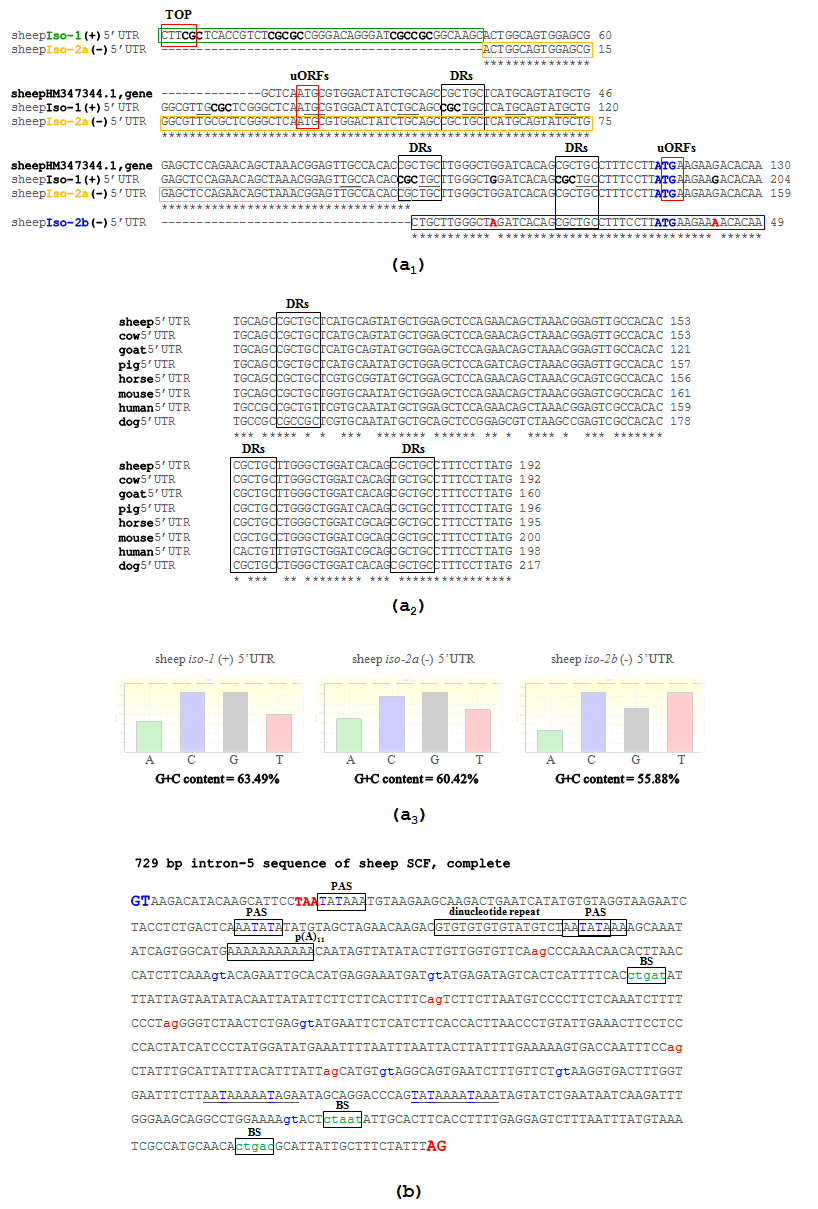


**Figure S4**


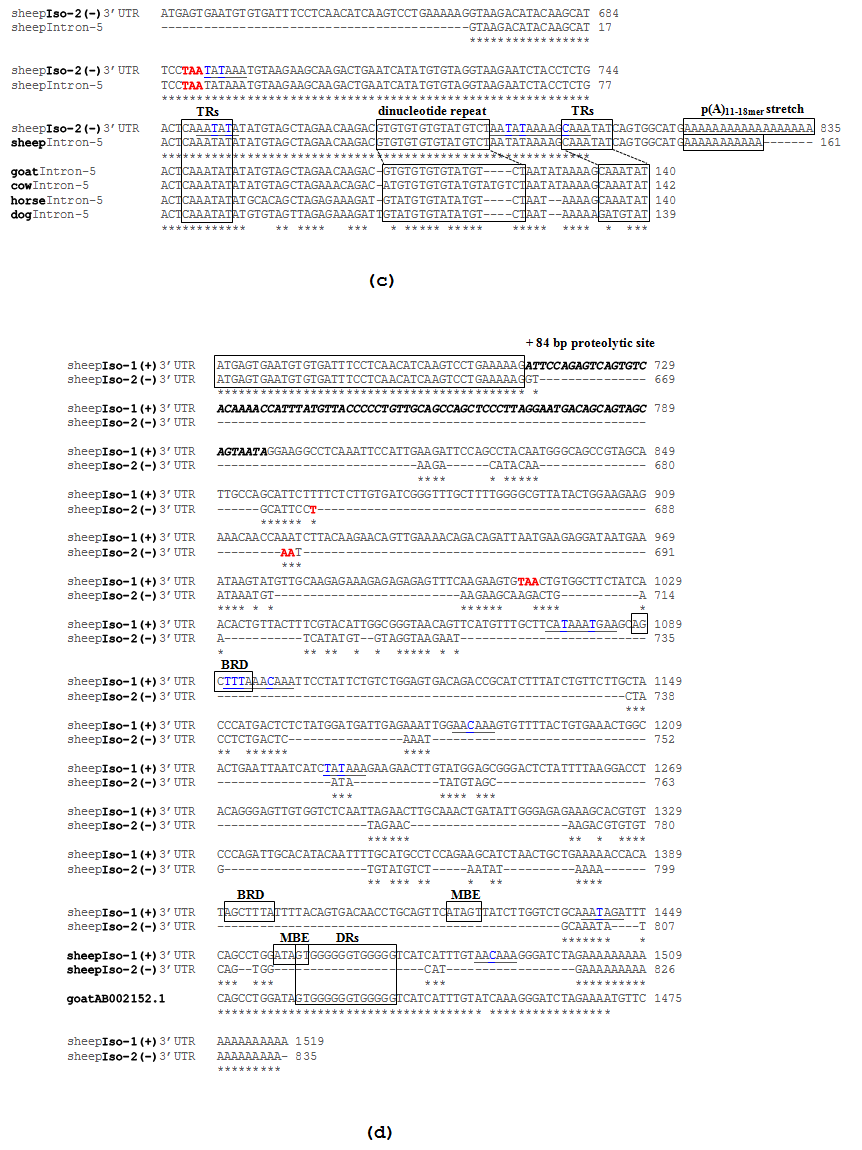

Supplement: Figure S4 — Nucleotide sequence comparison of 5′ and 3′ untranslated regions (UTRs) of sheep SCF isoform-1 (+) and isoform-2a/2b (−), the predicted UTR regulatory motifs and the possible splice donor/acceptor sites on intron-5 are shown. (a1) Sequence alignment shows sheep SCF 5′ UTR length differences between isoform-1 (+) and isoform-2a/2b (−). The additional sequences of 5′ UTRs are indicated in green, light orange and blue opened boxes for isoform-1 (+), isoform-2a (+) and isoform-2b (−), respectively. The cis-regulatory elements located in the the 5′ UTR such as TOP and uORFs are labeled and indicated in red opened boxes. The trinucleotide elements such as ‘CGC’ and ‘TGC’ are highlighted in bold black letters and by underline respectively. The hexamer direct repeats (DRs) are labeled and indicated by opened boxes. Clone differences are labeled in bold red to bold black letters; (a2) Alignment shows SCF 5′ UTR nucleotide sequence conservation of hexamer DRs (in opened boxes) with other mammals; (a3) Histogram shows the GC% of three different 5′ UTR of sheep SCF; (b) The complete sequence of sheep SCF intron-5 (729 bp) shows the constitutive splice donor (GT, in bold blue upper case letters) site at the start and the constitutive splice acceptor (AG, in bold red upper case letters) site at the end. Other alternative isoform/cryptic splice donor (gt), acceptor (ag) sites are labeled in blue, red lower case letters respectively. The dinucleotide repeats, polyA stretch (p(A)) and predicted splice branch sites (BS, in green lower case letters) are labeled and highlighted in opened boxes; (c) Nucleotide sequence alignment shows 100% similarity of 3′ UTR of isoform-2 (−) with 161 bp retained intron-5 of sheep SCF. The p(A) stretch and the conservation of dinucleotide repeats flanked by two tandem repeats (TRs) on either side of 3′ UTR are marked in opened boxes along with its counterpart sequences on intron-5 in other animals; (d) Sequence alignment shows two different 3′ UTRs of [file pone.0038657.s004.doc]

**Figure S5**


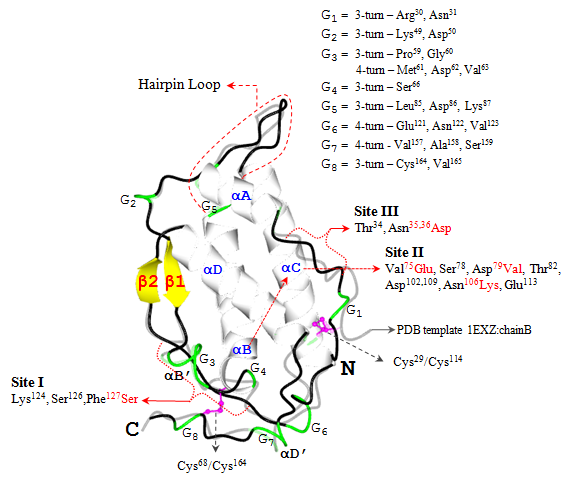

Supplement: Figure S5 — Three-dimensional structure of oSCF monomer generated by homology-based modelling represented by a ribbon diagram. Here the superimposition of oSCF monomer to the PDB template 1EXZ:chainB (set to transparency) is shown. The 4 α-helix, two antiparallel β-sheets, two additional one-turn helix are labelled in blue (αA, αB, αC and αD), red (β1, β2) and black (αB’, αD’) letters respectively. An exceptional hairpin loop between αB and αC is shown in red dotted line. The observed additional 3–4 turn helices are highlighted in green as G1 to G8 with the corresponding aa residues labeled respectively. The three potential interactive sites of SCF for its receptor c-kit are shown in bold letters as Site I, Site II and Site III [95]. In comparison to human SCF, the highlighted aa residue in red at Site I, II and III represents the orthologous substitution of aa residues in accordance with sheep, goat, cow, pig, dog, horse, cat and panda SCF to huSCF protein. The two disulfide bridges Cys29/Cys114 and Cys68/Cys164 are highlighted in pink. (DOC) [file pone.0038657.s005.doc]

**Figure S6**


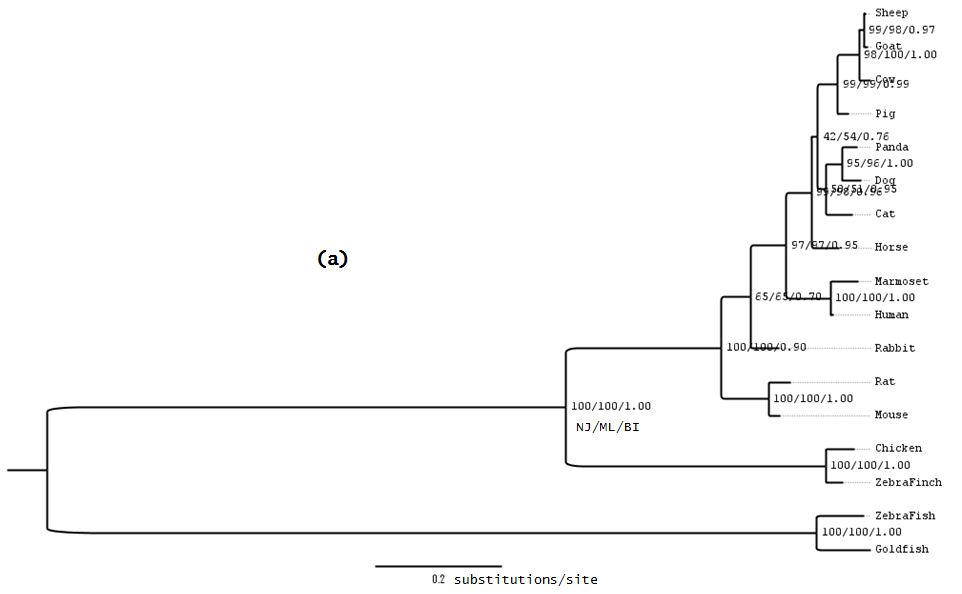


**Figure S6**


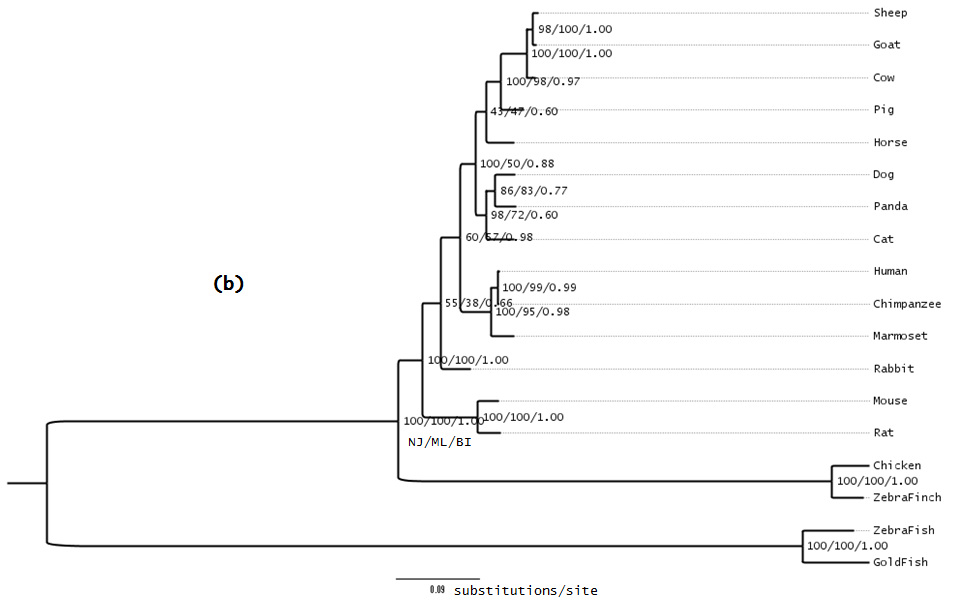


**Figure S6**


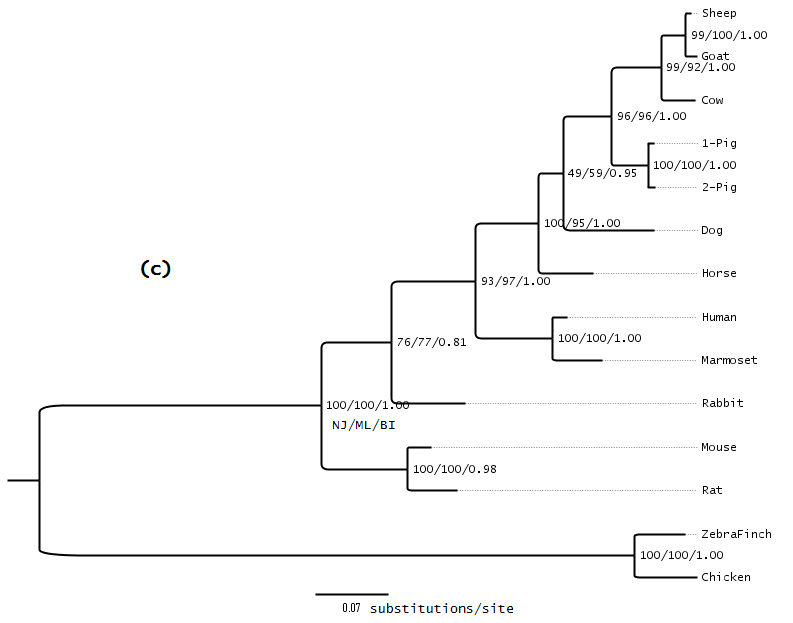


**Figure S6**


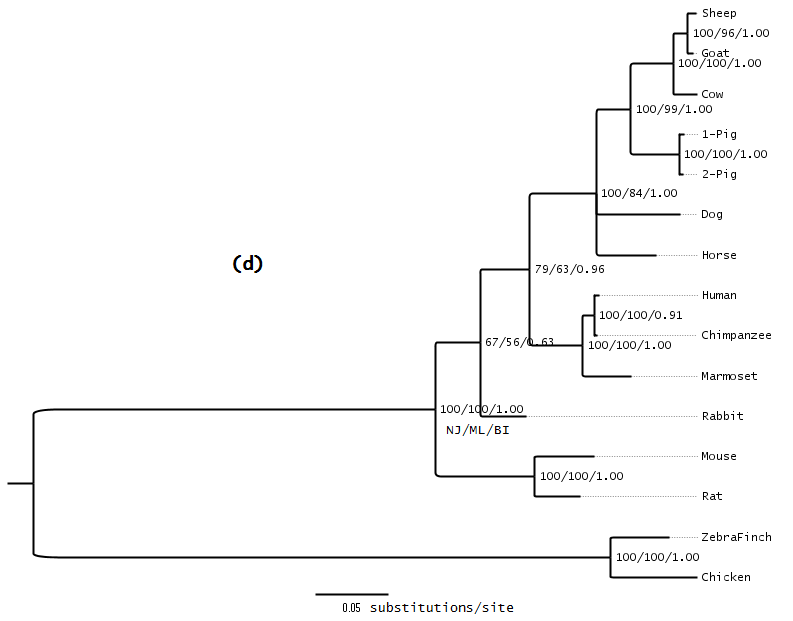


**Figure S6**


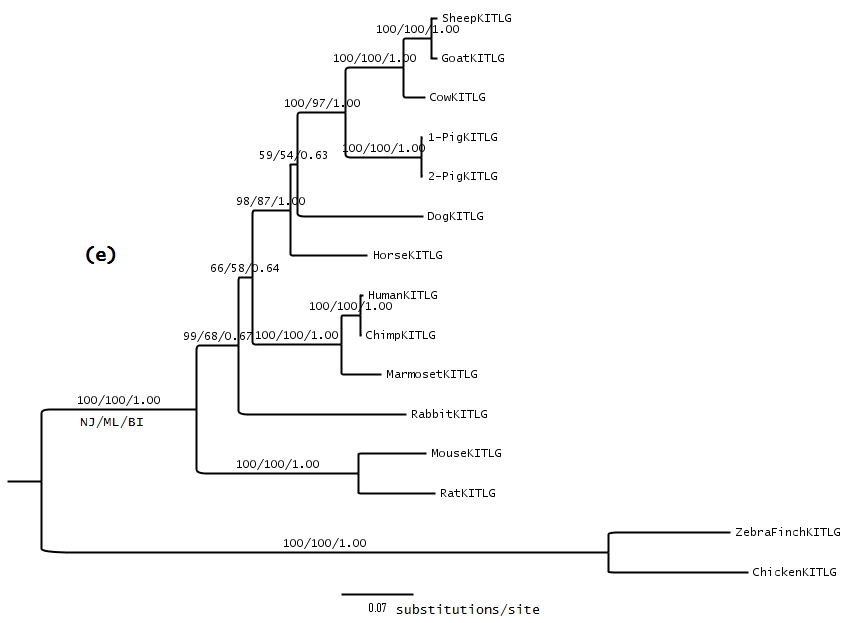

Supplement: Figure S6 — Phylogenetic analysis of the two SCF isoforms based on alignment of their complete nucleotide sequences (CDS), deduced amino acid sequences, and predicted DNA sequences representing exon(5)-intron-5-exon(6) splice junction of SCF gene. Numbers on the respective nodes denote percentages in the order of Neighbour-Joining (NJ) using p-distance/Maximum likelihood (ML)/Bayesian (BI) posterior probabilities. The values in the tree nodes represent bootstrap values of 1000 trials, indicating the credibility of each branch. Branch lengths are proportional to the number of amino acid or nucleotide changes on the branch. (a) Phylogenetic tree inferred from 17 soluble SCF (+) protein sequences; (b) Phylogenetic tree inferred from 18 soluble SCF (+) nucleotide sequences; (c) Phylogenetic tree inferred from 13 membrane-bound SCF (−) predicted protein sequences; (d) Phylogenetic tree inferred from 14 membrane-bound SCF (−) predicted nucleotide sequences; (e) Phylogenetic tree inferred from 14 predicted DNA sequences representing exon(5)-intron-5-6-exon(6) splice junction (+/−) of SCF gene. (DOC) [file pone.0038657.s006.doc]
