# Supplementary material for: Skipping of Exons by Premature Termination of Transcription and Alternative Splicing within Intron-5 of the Sheep SCF Gene: A Novel Splice Variant
Source: PLoS One. 2012 Jun 15;7(6):e38657. doi: 10.1371/journal.pone.0038657 (PMC3376141; doi:10.1371/journal.pone.0038657)
Supplement: Table S1 — GenBank Accession Nos. and description of ovine SCF cDNAs submitted to NCBI. (DOC) [file pone.0038657.s007.doc]

**Table S1. GenBank Accession Nos. and description of ovine SCF cDNAs submitted to NCBI**

| **GenBank Accession No.** | **Description** |
| --- | --- |
| GU386371 | *Ovis aries* stem cell factor (SCF), genomic DNA, partial cds, Exon(5)-Intron(5-6)-Exon(6) splice junction (Ref. HUMAN), Premature Intronic Stop Codon resulting in Truncated Transcript Variant-2a,b (-) form or alternatively spliced Transcript Variant-1, (+) form |
| GU386372 | *Ovis aries* stem cell factor (SCF), complete mRNA/cDNA, Transcript Variant-1, (+) form, has the primary proteolytic-cleavage site, soluble product |
| GU386373 | *Ovis aries* stem cell factor (SCF), complete mRNA/cDNA, Truncated, Transcript Variant-2a, (-) form, Premature Stop Codon, lacks the primary proteolytic-cleavage site, membrane-bound product, has 110 bp additional longer 5’ UTR seq. as compared to the shorter Transcript Variant-2b (34 bp) |
| GU386374 | *Ovis aries* stem cell factor (SCF), complete mRNA/cDNA, Transcript Variant-2b, (-) form, alternatively spliced, has 34 bp shorter, 5’ UTR seq. as compared to the longer 5’ UTR seq. of Transcript Variant-2a (144 bp) |
