# Supplementary material for: Skipping of Exons by Premature Termination of Transcription and Alternative Splicing within Intron-5 of the Sheep SCF Gene: A Novel Splice Variant
Source: PLoS One. 2012 Jun 15;7(6):e38657. doi: 10.1371/journal.pone.0038657 (PMC3376141; doi:10.1371/journal.pone.0038657)
Supplement: Table S2 — Details of Oligonucleotide primers used and the corresponding experiment/target amplification of ovine SCF isoforms. (DOC) [file pone.0038657.s008.doc]

**Table S2.** Details of Oligonucleotide primers used and the corresponding experiment/target amplification of ovine SCF isoforms

| **Target/Experiment** | **Primer set** | **Sequence (5’ 3’)** | **Tm (^o^C)** | **Ta (^o^C)** | **PCR product (bp)** |
| --- | --- | --- | --- | --- | --- |
| **coding sequences (CDS)** | scffwd1 (common*)  scfrev1 (+ form) | gcgctgcctttccttATGa  attactgctactgctgtcattcct | 65.2  61.9 | 61 | 621 |
| **3’ RACE amplification** | | | | |  |
| **(+) form**  Normal cDNA synthesis  cDNA synthesis, 3’R RT-PCR as a reverse primer  1^st^ round 3’R RT-PCR (common)  2^nd^ round 3’R RT-PCR (Nested 1) (+ form)  3^rd^ round 3’R RT-PCR (Nested 2) (+ form) | Oligo(dT) _18_  Oligo(dT)_18_modified  scf3’Rfwd1 (common)  stpro3’Rfwd1 (+ form)  stpro3’Rfwd2 (+ form) | (t)_18_  gagagagagagagacagagaactagtctcgag(t)_18_  gcgctgcctttccttATGaagaagacacaaact  tcaagtcctgaaaaagattccagagtcagtg  agccagctcccttaggaatg | 48.8  74.9  75.9  72.0  64.4 | 69/72  69  61 | 738/1377 (expected)  855  793 |
| **(-) form)**  cDNA synthesis, 3’ R RT-PCR as a reverse primer  1^st^ round 3’R RT-PCR (common)  2^nd^ round 3’R RT-PCR (Nested 1) (common)  3^rd^ round 3’R RT-PCR (Nested 2) (common)  4^th^ round 3’R RT-PCR (Nested 3) (common) | Oligo(dT) _18_modified  scf3’Rfwd1 (common)  scf3’Rfwd2 (common)  scf3’Rfwd3 (common)  scf3’Rfwd4 (common) | gagagagagagagacagagaactagtctcgag(t)_18_  gcgctgcctttccttATGaagaagacacaaact  ttggtggcaaatcttccaaaagactatatgataaccc  tggagtgcatggaagaacactcatttgagaatg  ccagaacccaggcagtttactc | 74.9  75.9  74.7  76.5  65.8 | 69/72  69/72  69/72  61 | 738/1377 (expected)  597  389  336 |
| **5’ RACE amplification** |  |  |  |  |  |
| **(+) form**  cDNA synthesis, 1^st^ round 5’R RT-PCR  2^nd^ round 5’R RT-PCR (Nested) | aapfwd (invitrogen)  auapfwdnst (invitrogen)  scfrev1 (+ form)  scfrev3 (common) | ggccacgcgtcgactagtacgggiigggiigggiig  ggccacgcgtcgactagtac  attactgctactgctgtcattcct  ggaagatttgccaccaattttgta | 83.2  66.9  61.9  66.4 | 61/57  61 | 364 (372) |
| **(-) form**  cDNA synthesis, 1^st^ round 5’R RT-PCR  2^nd^ round 5’R RT-PCR (Nested) | aapfwd (invitrogen)  auapfwdnst (invitrogen)  scfrev2 (common)  scfrev3 (common) | ggccacgcgtcgactagtacgggiigggiigggiig  ggccacgcgtcgactagtac  tcttccatgcactccacaag  ggaagatttgccaccaattttgta | 83.2  66.9  63.9  66.4 | 61  61 | 325 and 215 |
| **DNA splice junction amplification**  Exon 5-Intron (5-6)-Exon 6, premature termination (Ref. Genome human and mouse) | scffwd3 (common)  scfrev1 (+ form) | ccagaacccaggcagtttactc  attactgctactgctgtcattcct | 65.8  61.9 | 61 | 948 |
| **Semi-quantitativeRT-PCR** | | | | |  |
| **House keeping genes (HKGs)**  GeneBank Acc. No. AM711875.1|sheep, gene  NR_002170.3|pig, NR_003286.2|human, NR_036642.1|cow, NR_003278.1|mouse | ovine18S rRNAfwd  ovine18S rRNArev | gtgacgaaaaataacaatacagg  ctattggagctggaattacc | 59.0  57.7 | 53 | 132 (probe for Northern blot) |
| GeneBank Acc. No. NM_001190390.1|Sheep | ovineGAPDHfwd  ovineGAPDHrev | atcactgccacccagaagac  ctgcttcaccaccttcttga | 64.2  63.1 | 58 | 252 |
| **(+) form** (set-1) | stpro3’Rfwd1 (+ form)  scfrev1 (+ form) | tcaagtcctgaaaaagattccagagtcagtg  attactgctactgctgtcattcct | 72.0  61.9 | 57 | 99 |
| **(+) form** (set-2) | scffwd1 (common)  scfrev1 (+ form) | gcgctgcctttccttATGa  attactgctactgctgtcattcct | 65.2  61.9 | 61 | 621 |
| **(-) form** (set-3) | scffwd4 (common)  scf(-)rev (- form) | caaggacttggagatagtggcttc  ttaggaatgcttgtatgtcttacctttttcaggacttga | 65.9  73.9 | 61 | 94 |
| **(-) form** (set-4) | scf3’Rfwd3 (common)  scf(-)rev (- form) | tggagtgcatggaagaacactcatttgagaatg  ttaggaatgcttgtatgtcttacctttttcaggacttga | 76.5  73.9 | 69 | 212 |
| **(+/-) form** | scffwd2 (common) | ggtggcaaatcttccaaaag | 63.2 |  |  |
|  | scfrev2 (common) | tcttccatgcactccacaag | 63.9 | 58 | 222 (probe for Northern blot) |

* common --- represents common region in the CDS of the SCF (+) and (-) form except for ‘scffwd1’ which has 15 bp upstream 5’ UTR sequence plus 4 bp of the CDS including the start codon ATG.
