# Supplementary material for: Skipping of Exons by Premature Termination of Transcription and Alternative Splicing within Intron-5 of the Sheep SCF Gene: A Novel Splice Variant
Source: PLoS One. 2012 Jun 15;7(6):e38657. doi: 10.1371/journal.pone.0038657 (PMC3376141; doi:10.1371/journal.pone.0038657)
Supplement: Table S3 — (A) Molecular annotation of mRNAs, the pre-messenger or transcription unit, the 5 kb upstream and the UTRs for SCF (KITLG; source: Aceview*). (B) Comprehensive details of SCF mRNAs structure and different tissue expression (source: AceView). (C) Details of the validated polyA sites of SCF (source: AceView). (D) Details of the alternatively spliced, predicted SCF protein properties (source: AceView). (DOC) [file pone.0038657.s009.doc]

**Table S3A.** Molecular annotation of mRNAs, the pre-messenger or transcription unit, the 5 kb upstream and the UTRs for SCF (KITLG; source: Aceview*)

| **mRNA variant** | **mRNA matching the genome** | **Best predicted protein** | **5’ UTR** | **3’ UTR** | **Upstream sequence** | **Transcription unit pre-mRNA** | **Downstream sequence** |
| --- | --- | --- | --- | --- | --- | --- | --- |
| [aApr07](javascript:openAceViewLink('MRNA', 'KITLG.aApr07', 'Fiche') ;) | [5448 bp](javascript:openAceViewLink('DNA_mRNA::', 'KITLG.aApr07', 'DNA') ;) | [273 aa](javascript:openAceViewLink('PEP_Product::', 'KITLG.aApr07', 'Fiche') ;) | [196 bp](javascript:openAceViewLink('DNA_mRNA:1:196', 'KITLG.aApr07', 'DNA') ;) | [4430 bp](javascript:openAceViewLink('DNA_mRNA:1019:5448', 'KITLG.aApr07', 'DNA') ;) | [5kb including Promoter](javascript:openAceViewLink('DNA:651442:656441:0', 't12_Hs12_19702_36_20_t12_Hs12_19702_36_21', 'DNA') ;) | [87686 bp](javascript:openAceViewLink('DNA_mRNA:99999:99999', 'KITLG.aApr07', 'DNA') ;) | [1kb](javascript:openAceViewLink('DNA:568758:569757:0', 't12_Hs12_19702_36_20_t12_Hs12_19702_36_21', 'DNA') ;) |
| [bApr07](javascript:openAceViewLink('MRNA', 'KITLG.bApr07', 'Fiche') ;) | [5358 bp](javascript:openAceViewLink('DNA_mRNA::', 'KITLG.bApr07', 'DNA') ;) | [245 aa](javascript:openAceViewLink('PEP_Product::', 'KITLG.bApr07', 'Fiche') ;) | [190 bp](javascript:openAceViewLink('DNA_mRNA:1:190', 'KITLG.bApr07', 'DNA') ;) | [4430 bp](javascript:openAceViewLink('DNA_mRNA:929:5358', 'KITLG.bApr07', 'DNA') ;) | [5kb including Promoter](javascript:openAceViewLink('DNA:651436:656435:0', 't12_Hs12_19702_36_20_t12_Hs12_19702_36_21', 'DNA') ;) | [87680 bp](javascript:openAceViewLink('DNA_mRNA:99999:99999', 'KITLG.bApr07', 'DNA') ;) | [1kb](javascript:openAceViewLink('DNA:568758:569757:0', 't12_Hs12_19702_36_20_t12_Hs12_19702_36_21', 'DNA') ;) |
| [cApr07](javascript:openAceViewLink('MRNA', 'KITLG.cApr07', 'Fiche') ;) | [5194 bp](javascript:openAceViewLink('DNA_mRNA::', 'KITLG.cApr07', 'DNA') ;) | [238 aa](javascript:openAceViewLink('PEP_Product::', 'KITLG.cApr07', 'Fiche') ;) | [48 bp](javascript:openAceViewLink('DNA_mRNA:1:48', 'KITLG.cApr07', 'DNA') ;) | [4429 bp](javascript:openAceViewLink('DNA_mRNA:766:5194', 'KITLG.cApr07', 'DNA') ;) | [5kb possibly including promoter](javascript:openAceViewLink('DNA:605730:610729:0', 't12_Hs12_19702_36_20_t12_Hs12_19702_36_21', 'DNA') ;) | [41973 bp](javascript:openAceViewLink('DNA_mRNA:99999:99999', 'KITLG.cApr07', 'DNA') ;) | [1kb](javascript:openAceViewLink('DNA:568759:569758:0', 't12_Hs12_19702_36_20_t12_Hs12_19702_36_21', 'DNA') ;) |
| [dApr07-unspliced](javascript:openAceViewLink('MRNA', 'KITLG.dApr07-unspliced', 'Fiche') ;) | [3919 bp](javascript:openAceViewLink('DNA_mRNA::', 'KITLG.dApr07-unspliced', 'DNA') ;) | [139 aa](javascript:openAceViewLink('PEP_Product::', 'KITLG.dApr07', 'Fiche') ;) | [201 bp](javascript:openAceViewLink('DNA_mRNA:1:201', 'KITLG.dApr07-unspliced', 'DNA') ;) | [3298 bp](javascript:openAceViewLink('DNA_mRNA:622:3919', 'KITLG.dApr07-unspliced', 'DNA') ;) | [5kb including Promoter](javascript:openAceViewLink('DNA:576696:581695:0', 't12_Hs12_19702_36_20_t12_Hs12_19702_36_21', 'DNA') ;) | [3919 bp](javascript:openAceViewLink('DNA_mRNA:99999:99999', 'KITLG.dApr07-unspliced', 'DNA') ;) | [1kb](javascript:openAceViewLink('DNA:577779:578778:0', 't12_Hs12_19702_36_20_t12_Hs12_19702_36_21', 'DNA') ;) |
| [eApr07](javascript:openAceViewLink('MRNA', 'KITLG.eApr07', 'Fiche') ;) | [558 bp](javascript:openAceViewLink('DNA_mRNA::', 'KITLG.eApr07', 'DNA') ;) | [75 aa](javascript:openAceViewLink('PEP_Product::', 'KITLG.eApr07', 'Fiche') ;) | [190 bp](javascript:openAceViewLink('DNA_mRNA:1:190', 'KITLG.eApr07', 'DNA') ;) | [140 bp](javascript:openAceViewLink('DNA_mRNA:419:558', 'KITLG.eApr07', 'DNA') ;) | [5kb including Promoter](javascript:openAceViewLink('DNA:651436:656435:0', 't12_Hs12_19702_36_20_t12_Hs12_19702_36_21', 'DNA') ;) | [61620 bp](javascript:openAceViewLink('DNA_mRNA:99999:99999', 'KITLG.eApr07', 'DNA') ;) | [1kb](javascript:openAceViewLink('DNA:594818:595817:0', 't12_Hs12_19702_36_20_t12_Hs12_19702_36_21', 'DNA') ;) |
| [fApr07](javascript:openAceViewLink('MRNA', 'KITLG.fApr07', 'Fiche') ;) | [784 bp](javascript:openAceViewLink('DNA_mRNA::', 'KITLG.fApr07', 'DNA') ;) | [69 aa](javascript:openAceViewLink('PEP_Product::', 'KITLG.fApr07', 'Fiche') ;) | [192 bp](javascript:openAceViewLink('DNA_mRNA:1:192', 'KITLG.fApr07', 'DNA') ;) | [382 bp](javascript:openAceViewLink('DNA_mRNA:403:784', 'KITLG.fApr07', 'DNA') ;) | [5kb probably including promoter](javascript:openAceViewLink('DNA:651438:656437:0', 't12_Hs12_19702_36_20_t12_Hs12_19702_36_21', 'DNA') ;) | [64137 bp](javascript:openAceViewLink('DNA_mRNA:99999:99999', 'KITLG.fApr07', 'DNA') ;) | [1kb](javascript:openAceViewLink('DNA:592303:593302:0', 't12_Hs12_19702_36_20_t12_Hs12_19702_36_21', 'DNA') ;) |
| [gApr07](javascript:openAceViewLink('MRNA', 'KITLG.gApr07', 'Fiche') ;) | [568 bp](javascript:openAceViewLink('DNA_mRNA::', 'KITLG.gApr07', 'DNA') ;) | [56 aa](javascript:openAceViewLink('PEP_Product::', 'KITLG.gApr07', 'Fiche') ;) | [258 bp](javascript:openAceViewLink('DNA_mRNA:1:258', 'KITLG.gApr07', 'DNA') ;) | [139 bp](javascript:openAceViewLink('DNA_mRNA:430:568', 'KITLG.gApr07', 'DNA') ;) | [5kb including Promoter](javascript:openAceViewLink('DNA:651442:656441:0', 't12_Hs12_19702_36_20_t12_Hs12_19702_36_21', 'DNA') ;) | [83395 bp](javascript:openAceViewLink('DNA_mRNA:99999:99999', 'KITLG.gApr07', 'DNA') ;) | [1kb](javascript:openAceViewLink('DNA:573049:574048:0', 't12_Hs12_19702_36_20_t12_Hs12_19702_36_21', 'DNA') ;) |
| [hApr07](javascript:openAceViewLink('MRNA', 'KITLG.hApr07', 'Fiche') ;) | [553 bp](javascript:openAceViewLink('DNA_mRNA::', 'KITLG.hApr07', 'DNA') ;) | [37 aa](javascript:openAceViewLink('PEP_Product::', 'KITLG.hApr07', 'Fiche') ;) | [79 bp](javascript:openAceViewLink('DNA_mRNA:1:79', 'KITLG.hApr07', 'DNA') ;) | [360 bp](javascript:openAceViewLink('DNA_mRNA:194:553', 'KITLG.hApr07', 'DNA') ;) | [5kb including Promoter](javascript:openAceViewLink('DNA:651435:656434:0', 't12_Hs12_19702_36_20_t12_Hs12_19702_36_21', 'DNA') ;) | [61635 bp](javascript:openAceViewLink('DNA_mRNA:99999:99999', 'KITLG.hApr07', 'DNA') ;) | [1kb](javascript:openAceViewLink('DNA:594802:595801:0', 't12_Hs12_19702_36_20_t12_Hs12_19702_36_21', 'DNA') ;) |

* The AceView mRNA models are a non-redundant, comprehensive and curated representation of the cDNA sequence data in the public repositories (GenBank and dbEST). This table documents the physical properties of each representative mRNA, whose sequence has been corrected for sequencing errors and matched to the genome.

**Table S3B.** Comprehensive details of SCF mRNAs structure and different tissue expression (source: AceView)

| **mRNA variant** | **No. of exons** | **No. of clones** | **From tissue (no strict specificity is implied)** | **5’ completeness evidence** | **3’ completeness evidence** | **coordinates on gene** |
| --- | --- | --- | --- | --- | --- | --- |
| [aApr07](javascript:openAceViewLink('MRNA', 'KITLG.aApr07', 'Fiche') ;) | 10 | 38 | testis (4), trachea (4), brain (3), small intestine (2) and 12 other tissues | capped | validated polyA | 1 to 87686 |
| [bApr07](javascript:openAceViewLink('MRNA', 'KITLG.bApr07', 'Fiche') ;) | 9 | 112 | brain (10), trachea (9), whole brain (7), thalamus (6) and 49 other tissues | capped | validated polyA | 7 to 87686 |
| [cApr07](javascript:openAceViewLink('MRNA', 'KITLG.cApr07', 'Fiche') ;) | 9 | 23 | placenta (5), lung (3), uterus (3), well-differentiated endometrial adenocarcinoma, 7 pooled tumors (3) and 21 other tissues | 5’ stop | validated polyA | 45713 to 87685 |
| [dApr07-unspliced](javascript:openAceViewLink('MRNA', 'KITLG.dApr07-unspliced', 'Fiche') ;) | 1 | 10 | placenta (2) | capped | validated polyA | 74747 to 78665 |
| [eApr07](javascript:openAceViewLink('MRNA', 'KITLG.eApr07', 'Fiche') ;) | 5 | 1 | kidney, tumor tissue (1) | capped | 3’ stop | 7 to 61626 |
| [fApr07](javascript:openAceViewLink('MRNA', 'KITLG.fApr07', 'Fiche') ;) | 5 | 4 | prostate (1) | aggregated clones | 3’ stop | 5 to 64141 |
| [gApr07](javascript:openAceViewLink('MRNA', 'KITLG.gApr07', 'Fiche') ;) | 5 | 1 |  | capped | 3’ stop | 1 to 83395 |
| [hApr07](javascript:openAceViewLink('MRNA', 'KITLG.hApr07', 'Fiche') ;) | 5 | 1 | kidney (1) | capped | No evidence | 8 to 61642 |

**Table S3C.** Details of the validated polyA sites of SCF (source: AceView)

| **Position on the gene** | **Position on the mRNA** | **Number of supporting accessions** | **Poly-A signal** | **Distance to poly-A site** |
| --- | --- | --- | --- | --- |
| 83655 | bp 1417 on .a | 2 | Variant | 38 |
| 87542 | bp 5214 on .b | 10 | AATAAA | 22 |
| 87681 | bp 5353 on .b | 1 | AATAAA | 22 |
| 87681 | bp 5190 on .c | 21 | AATAAA | 22 |
| 78632 | bp 3886 on .d-u | 1 | Variant | 25 |

**Table S3D.** Details of the alternatively spliced, predicted SCF protein properties (source: AceView)

| **Protein** | **Protein quality** | **Exons in CDS** | **Domains** | **Predicted localization** | **Completeness and uniqueness** | **Extends from** | **coordinates on mRNA** | **minimal set of supporting clones*** |
| --- | --- | --- | --- | --- | --- | --- | --- | --- |
| [.a](javascript:openAceViewLink('MRNA', 'KITLG.aApr07', 'Fiche') ;) | 273 aa Very good | 9 | Stem cell factor, coiled coil stretch | nuclear | complete | Met (ATG) to Stop | 197 to 1018 | [BC126166](javascript:openAceViewLink('Sequence', 'BC126166', 'Fiche') ;) |
| [.b](javascript:openAceViewLink('MRNA', 'KITLG.bApr07', 'Fiche') ;) | 245 aa Very good | 8 | Stem cell factor, coiled coil stretch |  | complete | Met (ATG) to Stop | 191 to 928 | [AF119835](javascript:openAceViewLink('Sequence', 'AF119835', 'Fiche') ;) |
| [.c](javascript:openAceViewLink('MRNA', 'KITLG.cApr07', 'Fiche') ;) | 238 aa Very good | 8 | Stem cell factor, coiled coil stretch | nuclear | complete | Met (ATG) to Stop | 49 to 765 | [CR749222](javascript:openAceViewLink('Sequence', 'CR749222', 'Fiche') ;) |
| [.d](javascript:openAceViewLink('MRNA', 'KITLG.dApr07-unspliced', 'Fiche') ;) | 139 aa Good | 1 |  |  | complete | Met (ATG) to Stop | 202 to 621 | [AK127865](javascript:openAceViewLink('Sequence', 'AK127865', 'Fiche') ;) |
| [.e](javascript:openAceViewLink('MRNA', 'KITLG.eApr07', 'Fiche') ;) | 75 aa Very good | 4 | Stem cell factor | cytoplasmic | complete | Met (ATG) to Stop | 191 to 418 | [DB173336](javascript:openAceViewLink('Sequence', 'DB173336', 'Fiche') ;) |
| [.f](javascript:openAceViewLink('MRNA', 'KITLG.fApr07', 'Fiche') ;) | 69 aa Good | 3 | Stem cell factor |  | complete | Met (ATG) to Stop | 193 to 402 | [BF675585](javascript:openAceViewLink('Sequence', 'BF675585', 'Fiche') ;) |
| [.g](javascript:openAceViewLink('MRNA', 'KITLG.gApr07', 'Fiche') ;) | 56 aa Good | 3 | Stem cell factor, coiled coil stretch |  | complete included in aApr07, bApr07, cApr07 | Met (ATG) to Stop | 259 to 429 | [DA648788](javascript:openAceViewLink('Sequence', 'DA648788', 'Fiche') ;) |
| [.h](javascript:openAceViewLink('MRNA', 'KITLG.hApr07', 'Fiche') ;) | 37 aa Apparently non coding | 1 |  |  | complete | Met (ATG) to Stop | 80 to 193 | [DA625716](javascript:openAceViewLink('Sequence', 'DA625716', 'Fiche') ;) |

* The above table allows to see at a glance from the last column if an isoform has its exonic structure fully supported by a single clone (the variant identifier a, b, c under such mRNA is underlined in the gene diagram in Figure S2), or if it requires concatenation of two or more cDNA clones (identifier not underlined in Figure S2).
